# Membrane-targeted push-pull azobenzenes for the optical modulation of membrane potential

**DOI:** 10.1038/s41377-024-01669-x

**Published:** 2025-01-01

**Authors:** Valentina Sesti, Arianna Magni, Matteo Moschetta, Chiara Florindi, Marlene E. Pfeffer, Mattia Lorenzo DiFrancesco, Michele Guizzardi, Giulia Folpini, Luca Sala, Alessandra Gilda Ritacca, Beatrice Campanelli, Paola Moretti, Giuseppe Maria Paternò, Luca Maragliano, Matteo Tommasini, Francesco Lodola, Elisabetta Colombo, Fabio Benfenati, Chiara Bertarelli, Guglielmo Lanzani

**Affiliations:** 1 Department of Chemistry, Materials and Chemical Engineering, “Giulio Natta” Politecnico di Milano, Milano, 20133 Italy; 2 Center for Nanoscience and Technology, Istituto Italiano di Tecnologia, Milano, 20134 Italy; 3 Department of Physics, Politecnico di Milano, Milano, 20133 Italy; 4 Department of Biotechnology and Biosciences, University of Milano-Bicocca, Milano, 20126 Italy; 5 Center for Synaptic Neuroscience and Technology, Istituto Italiano di Tecnologia, Genova, 16132 Italy; 6 IRCCS Ospedale Policlinico San Martino, Genova, 16132 Italy; 7 Institute for Photonics and Nanotechnologies (IFN), National Research Council (CNR), Milano, 20133 Italy; 8 Istituto Auxologico Italiano IRCCS, Center for Cardiac Arrhythmias of Genetic Origin and Laboratory of Cardiovascular Genetics, Milano, 20095 Italy; 9 Department of Life and Environmental Sciences, Polytechnic University of Marche, Ancona, 60131 Italy; 10 Present Address: Department of Materials Science and Engineering, Stanford University, Stanford, CA 94305 USA

**Keywords:** Optics and photonics, Biomaterials - cells

## Abstract

We introduce a family of membrane-targeted azobenzenes (MTs) with a push-pull character as a new tool for cell stimulation. These molecules are water soluble and spontaneously partition in the cell membrane. Upon light irradiation, they isomerize from *trans* to *cis*, changing the local charge distribution and thus stimulating the cell response. Specifically, MTs photoisomerization induces clear and reproducible depolarization. The most promising species, MTP2, was extensively studied. Time-resolved spectroscopy techniques provide insights into the excited state evolution and a complete understanding of its isomerization reaction. Molecular Dynamics simulations reveal the spontaneous and stable partitioning of the compound into the cellular membrane, without significant alterations to the bilayer thickness. MTP2 was tested in different cell types, including HEK293T cells, primary neurons, and cardiomyocytes, and a steady depolarization is always recorded. The observed membrane potential modulation in in-vitro models is attributed to the variation in membrane surface charge, resulting from the light-driven modulation of the MT dipole moment within the cell membrane. Additionally, a developed mathematical model successfully captures the temporal evolution of the membrane potential upon photostimulation. Despite being insufficient for triggering action potentials, the rapid light-induced depolarization holds potential applications, particularly in cardiac electrophysiology. Low-intensity optical stimulation with these modulators could influence cardiac electrical activity, demonstrating potential efficacy in destabilizing and terminating cardiac arrhythmias. We anticipate the MTs approach to find applications in neuroscience, biomedicine, and biophotonics, providing a tool for modulating cell physiology without genetic interventions.

## Graphical abstract

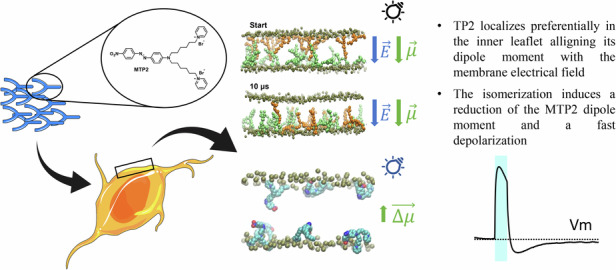

## Introduction

Light is a clean, non-invasive, and spatio-temporally precise tool for modulating a variety of biochemical functions in living cells, provided that the cells can absorb light. In most cases, this inherent light sensitivity is lacking, prompting the exploration of various approaches to confer this capability^1–4^. One paradigm involves the use of photochromic transducers, with azobenzenes (ABs) standing out as the most prominent example^5^. The AB isomerization reaction from the stable *trans* to the metastable *cis* form can be exploited to modulate the activity of proteins, nucleic acids, and ion channels in a light-controlled fashion^6–8^. To enhance the potential of ABs in precisely and remotely regulating specific aspects of cellular functioning, considerable efforts have been directed toward optimizing (i) their compatibility with the biological environment, (ii) their spectral response, (iii) their switching behaviour and (iv) cell compartments targeting. Desirable features for these applications include water solubility, reversible *trans–cis* isomerization reaction, and spectral operation in the visible-NIR region of the spectrum to avoid interfering with cell viability.

The use of ABs in photostimulation mostly relies on the engineering of light-gated transducers, showcasing the ability to sensitize endogenous proteins to light without the need for genetic modification. The latest iterations of these photoswitches are responsive to visible light and can induce reversible blocking of voltage- or ligand-gated ion channels intracellularly at the channel site^9^. However, these compounds are limited in their ability to produce negative contrast information and can pose invasiveness issues, due to the direct interaction with active components of the cell such as proteins, possibly hampering translation applications.

As an alternative, research started looking into molecular photoactuators, which spontaneously partition within the cell membrane and can transduce a light stimulus into a perturbation that can be detected and processed by cells. In this framework in the past few years, we developed an amphiphilic AB, namely Ziapin2^10–13^ which allows photostimulation in a less invasive way compared to current methods. The cationic terminal groups of Ziapin2 interact with the polar heads of phospholipids in the membrane, while the hydrophobic portion of the molecule stabilizes within the lipidic region. We have demonstrated that upon illumination, Ziapin2 modulates neuronal membrane potential and firing both in vitro and in vivo. In dark conditions, Ziapin2 molecules sitting in the two opposite membrane leaflets form transmembrane dimers, which reduce the membrane thickness and increase its electrical capacitance. Under light (470 nm), *trans–cis* photo-isomerization breaks the dimers, leading to the relaxation of the membrane back to its initial thickness, thereby reducing its capacitance. This opto-mechanical effect results in a transient hyperpolarization of the cell membrane potential followed by depolarization, without affecting local temperature^10^. While Ziapin2 has demonstrated wide and effective applicability for the photomodulation of the membrane potential in various cell types, including neurons, cardiac and skeletal muscle cells, and even in bacteria^14–17^, at least two challenges persist, namely, the poor solubility in water and the lack of a true off/on switching behaviour. Indeed, Ziapin2 already perturbs the membrane properties under dark conditions, exhibiting the typical behaviour of amphiphilic molecules that cause membrane thinning/thickening upon insertion in the membrane^18,19^ thus resulting in a biological event (i.e., the opening of mechano-sensitive channels^17,18^) in the absence of exogenous stimuli. Therefore, a fine balance between water solubility, the affinity of the molecule for the membrane environment and its dark-state activity would provide a more general rationale for the precise photomodulation of cell physiology. Comparable opto-capacitive effects have also been observed in a recent publication, in which azobenzenes have been inserted in the acyl chains of diacylglycerols (OptoDArG)^19^.

Here, we introduce new tools for cell stimulation, namely intramembrane ABs with a push–pull character. These molecules are water soluble and pioneer a different photostimulation paradigm, that is the light-induced modulation of the plasma membrane surface charge. We synthesized a series of new ABs, having in common the electron acceptor group NO_2_, and varying numbers of alkyl chains capped with cationic groups. The latter serves a dual role as membrane anchors and electron-donor groups. After the experimental screening of all the molecules in the series, based on their ability to partition into the membrane and induce light-dependent membrane potential changes, we selected the best candidate for physiological applications, namely MTP2. Through optical spectroscopy, we assessed the push–pull character of this molecule and its ability to undergo isomerisation in the biological setting. Subsequently, we evaluated its biological activity in terms of light-induced depolarization. Finally, we propose a model that aims to recapitulate the new MTP2 photostimulation mechanism.

## Results

### Molecular design and synthesis

**Scheme 1:**
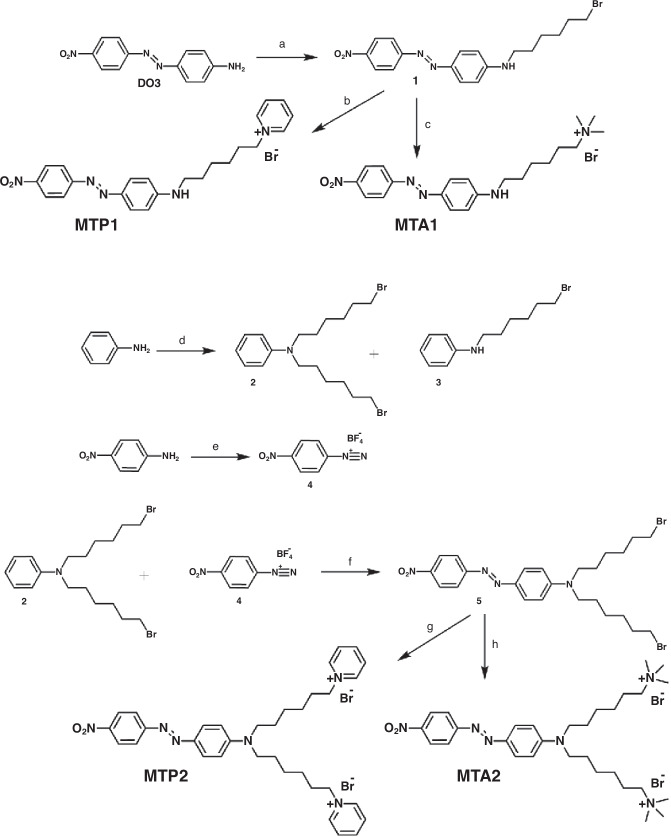
Synthesis of MTP1, MTA1, MTP2 and MTA2. **a** K_2_CO_3_ and 1,6-dibromohexane, CH_3_CN, 60 °C, 120 h, Ar; **b** Pyridine, room temperature, 120 h; **c** TMA, EtOH, 80 °C, 48 h; **d** 1,6-dibromohexane, 95 °C, 10 h, Ar; **e** BF_3_·EtOH and (CH_3_)_3_CONO, THF, −20 °C, 1 h; **f** CH_3_CN, 5 °C, 3 h; **g** Pyridine, room temperature, 24 h; **h** TMA, EtOH, 80 °C, 48 h

We collectively refer to the Membrane-Targeting push–pull ABs synthesized in this work as MTs. The replacement of the lipophilic azepane unit of our previously reported aminoazobenzenes^20^ with the strong electron withdrawing NO_2_ group leads to dramatic changes in the chemical and photophysical properties^21^. Four compounds were synthesized, which differ by the number of ω-substituted alkyl chains and the type of end-groups (Scheme 1). The monosubstituted MTs are obtained by alkylation of the amine of the Disperse Orange 3 (1 equiv.) with an excess α,ω-dibromohexane (2.2 equiv.) in acetonitrile under argon atmosphere (Scheme 1). The reaction was monitored through thin layer chromatography, showing the formation of the monosubstituted compound only (MTA1 and MTP1). This is ascribed to the deactivating effect of the nitro group, which is a strong acceptor. To obtain the disubstituted push–pull cationic terminated azobenzenes (MTA2 and MTP2), the azo-coupling between the diazonium salt of nitrobenzene (4) and the N,N-di-ω-bromohexyl aniline (2) was performed (Scheme 1). Details on the synthetic procedures and chemical characterization are reported in the Supplementary Materials and Methods.

In the Supplementary Materials, we report the results of the photophysical characterization of the MTs series. We confirmed that the push–pull character of these membrane-targeted azobenzenes (MTs) increases their solubility in water and leads to a red shift in the absorption with respect to the previously reported aABs^20^. The absorption spectra highlight that an increasing number of polar heads allows for an even higher solubility in water (Fig. S1), and they also show that the dialkylated MTs (MTA2 and MTP2) are more redshifted than the monosubstituted species (MTA1 and MTP1) (Fig. S2). Moreover, when the ability to spontaneously interact with the membrane was evaluated by a cell partitioning assay, a stronger affinity of MTP2 with the cell membrane was observed (Fig. S3). For all these reasons, we focused the following study on MTP2.

### Photophysical characterization of MTP2

We employed optical spectroscopy techniques to assess the interaction of MTP2 with the membrane environment. For this, we used water, as it is the solvent of choice for the biological application, and a water suspension of sodium dodecyl sulfate (SDS) micelles [100 mM], which represents a simple membrane-mimicking medium^22^. The photophysical features (peak position and featureless band shape) are coherent with push–pull azobenzenes highly reported in the literature^21^.

**Fig. 1:**
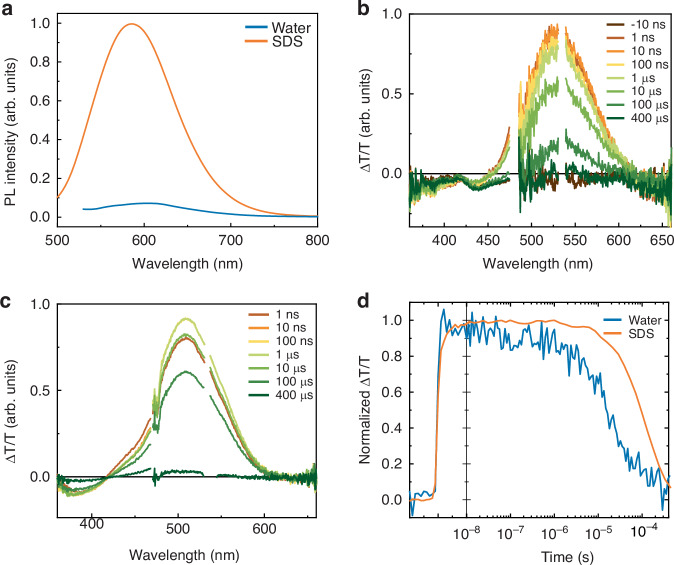
Steady-state and transient spectroscopy on MTP2 in water and SDS micelles suspension. **a** PL spectra of MTP2 [25 μM] in water and SDS micelles suspension, obtained by exciting the samples at the *λ*_max_, i.e. at 510 and 480 nm for water and SDS, respectively. **b**, **c** ms-transient absorption spectra of MTP2 in water (**b**) and SDS micellar suspension (**c**) at various delays. Spectra were collected by exciting the samples at 532 nm. **d** Dynamics of the GSB signal (520 and 500 nm for water and SDS, respectively) in the two experimental conditions

The steady-state photoluminescence (PL) spectrum shows a bathochromic shift from SDS to water, which can be assigned to the charge-transfer character of the first *trans*-isomer excited state (Fig. 1a), and the consequent reduction of the HOMO-LUMO gap with increasing solvent polarity. In addition, we observed that the relative emission yield is smaller in H_2_O than in SDS. For push–pull azobenzenes, we must also consider that the PL amplitude depends on two competitive non-radiative phenomena^12,23^: (i) the isomerization from *trans* to *cis*, which sweeps the population out of the Franck–Condon region where the radiative transition occurs. Accordingly, the emitting *trans*-steady-state population fraction is given by the equation ${n}_{{trans}}=\frac{{k}_{{CT}}{I}_{0}+\gamma }{{k}_{{TC}}{I}_{0}}{n}_{{cis}}$ where *k*_*CT*_ and *k*_*TC*_ are the light induced rates of *cis-trans* and *trans-cis* reactions, *γ* is the thermal back transfer from *cis* while ${I}_{0}$ is the CW light intensity; (ii) the internal conversions that thermally relaxes the excited state population to the ground state, affecting the PL quantum yield ($\eta$) of the *trans* isomer transition in the Frank Condon region, $\eta =\frac{{k}_{R}}{{k}_{R}+{k}_{{NR}}}$ where *k*_*R*_ and *k*_*NR*_ are the radiative and non-radiative decay rates, respectively. Both non-radiative decay paths depend on the interaction with the medium but in different ways.

To gain further insights into the photoisomerization dynamics of MTP2, we performed transient absorption (TA) measurements in the sub-nanosecond to millisecond time scale. Briefly, in TA spectroscopy, a short, intense pump laser pulse excites the sample, promoting electrons from the ground state to an excited state. Following a controlled delay, a second probe laser pulse measures the sample’s absorption at different time intervals. By varying the delay between the pump and probe pulses, the evolution of these excited states over time can be monitored. Transient absorption spectra usually show positive features, which correspond to either photobleaching (PB)—where the ground state is depleted, resulting in increased probe transmission—or to stimulated emission (SE), where photon emission gives the appearance of increased transmission. Conversely, negative features represent photoinduced absorption (PIA), where absorption from excited states reduces the probe transmission.

In the sub-ns-TA spectra (Figs. S4 and S5), we observe a positive ΔT/T signal, peaking around 500 nm, that overlaps the static absorbance of the molecule (Fig. S6) and is therefore associated with the ground state bleaching (GSB), and a negative ΔT/T signal assigned to PIA. We observed a fast dynamic component, completed in about 10 ps (fitting provides 2.7 ± 0.1 ps and 2.2 ± 0.05 ps in water and SDS, respectively) followed by a long-lived signal only seen in the GSB spectral region. We assign the first to *trans–cis* isomerization, and the latter to the depleted *trans* population. These experiments show that in both media MTP2 photoisomerizes, possibly with a larger yield of *cis* isomer in SDS as suggested by the larger amplitude of the long-lived plateau. To directly detect the *cis* isomer population, we extended TA measurements to the millisecond time regime. Transient spectra (Fig. 1b, c) show again a positive signal centred at 500 nm corresponding to the GSB signal and a negative signal below 400 nm assigned to the absorption of the *cis* isomer of MTP2. The estimates of the *cis* isomer lifetimes, obtained by fitting the dynamics of the GSB band (Fig. 1d) were 42 ± 4 μs and 300 ± 60 μs in water and SDS, respectively. Note that the *cis* lifetime for SDS micelles is an average between molecules in the micelles and molecules dispersed in water.

To summarize, following photoexcitation MTP2 isomerizes in about 10 ps, with a slightly larger yield and a sixfold longer lifetime of the cis isomer in SDS (300 μs) compared to water. Hence, the relatively higher PL amplitude in SDS compared to water is not due to a reduced isomerization rate, but to the hindering of non-radiative pathways caused by interactions with the micelle environment, due to increasing microviscosity and decreasing polarity, as reported previously for push–pull azobenzene molecules^24^.

### Molecular dynamics simulations of MTP2 partition into the cell membrane

**Fig. 2:**
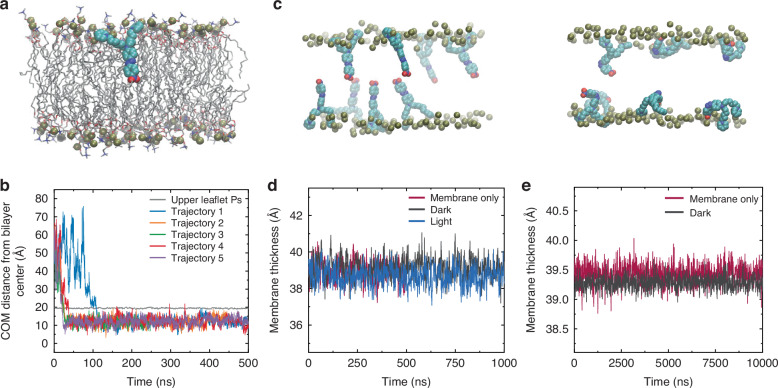
MTP2 spontaneously partitions into the lipid bilayer. **a** Snapshot extracted from an MD simulation showing a trans MTP2 (cyan) embedded into the membrane (POPC lipid model); lipid phosphorus atoms are shown as tan spheres, while water molecules are not reported for clarity. **b** Time-dependence of the distance between the centre of mass (COM) of MTP2 and the bilayer centre, in five independent MD trajectories; the grey line is the average position of the upper leaflet lipid head groups. **c** Snapshots extracted from the MD simulations of 8 MTP2 molecules (trans and cis) embedded in the membrane; water molecules and lipid acyl chains are not reported for clarity. **d** Time-dependence of the average thickness of membranes with zero (red line), eight trans (gray line), or eight cis (blue line) MTP2. **e** Time-dependence of the average bilayer thickness along the CG-MD simulations of solvated membranes with zero (red line) or 120 trans MTP2 molecules (black line)

To investigate the spontaneous insertion of MTP2 into the cellular membrane, we conducted five independent, 500 ns-long, atom-detailed Molecular Dynamics (MD) simulations. All trajectories started with a single MTP2 in the dark state at a random orientation within bulk water. In all instances, the molecule entered the bilayer before 200 ns (Fig. 2b), by first inserting its nitro-group moiety. It then remained embedded throughout the simulated trajectory, with the positively charged pyridine rings coordinated by the lipid heads phosphate groups (Fig. 2a). Inside the membrane, MTP2 maintained its longitudinal axis mostly parallel to the bilayer’s normal (Fig. S7), and no trans-bilayer movement was observed. We then simulated for 1 µs the behaviour of MTP2 molecules within a solvated membrane, distributed four per leaflet, separately in *trans* (dark) and *cis* (light) states (Fig. 2c). Again, no MTP2 molecule was seen to leave the bilayer during the simulated time interval. Also, no interaction between molecules from opposing leaflets was observed, resulting in no discernible modulation of the membrane thickness (Fig. 2d). Finally, to explore larger space and time scales, a coarse-grained (CG) system of 120 *trans*-MTP2 in a 270 × 270 Å^2^ POPC solvated membrane (Fig. S8) was simulated for 10 µs. Once more, no significant changes in the bilayer thickness were found when compared to a solvated membrane of the same size that does not contain MTP2 (Fig. 2e).

### Light-evoked modulation of the membrane potential in HEK293T cells

To study the possible interaction between MTP2 and the biological matter, we used the human embryonic kidney (HEK293T) cell line as a biological model for the initial characterization of the compound.

Firstly, the proliferation of MTP2-loaded HEK293T cells was assessed through AlamarBlue viability assay. Cell viability was evaluated up to 96 h after incubation with two concentrations of MTP2 (5 and 10 μM). Half of the samples were also exposed to 470 nm light at 200 μWmm^−2^ continuously for 30 s to exclude phototoxic effects. The assay does not show any significant change in cell viability/proliferation under both dark and light conditions (Fig. S9).

**Fig. 3:**
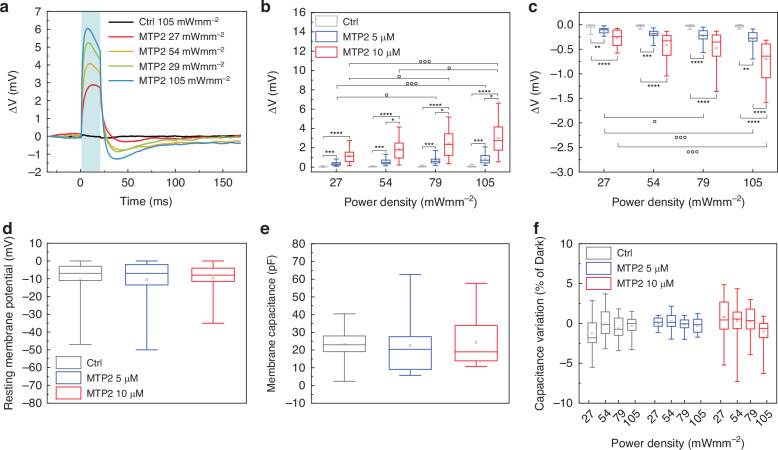
MTP2 depolarizes the membrane potential of HEK293T cells upon light stimulation. **a** Representative whole-cell current clamp traces recorded from HEK293T cells incubated for 5 min in the presence of either vehicle (Ctrl; black) or of MTP2 (10 µM; colour) and illuminated for 20 ms at increasing power densities. Traces were zeroed for presentation purposes. **b**, **c** Box plots of peak depolarization (**b**) and peak hyperpolarization (**c**) in HEK293T cells subjected to 20 ms of light stimulation in the presence of either vehicle (Ctrl; grey boxes) or of MTP2 at 5 and 10 µM (blue and red boxes, respectively). Cells were exposed at different power densities (27, 54, 79, and 105 mWmm^−2^). °,*p < 0.05; **p < 0.01; °°°,***p < 0.001; ****p < 0.0001; one-way ANOVA/Holm-Sidak’s tests or Kruskal–Wallis/Dunn’s tests (**b**: *n* = 21, 19, and 20; **c**: 21, 18, and 19; for Ctrl, MTP2 5 µM and MTP2 10 µM, respectively) ° refers to the comparison of the different power densities within the same concentration; * refers to the comparison between different concentrations. **d**, **e** Box plots representing resting membrane potential (**d**) and membrane capacitance (**e**) in HEK293T cells loaded with MTP2 for 5 min at 5 and 10 µM. **f** Box plots representing the changes in capacitance upon irradiation at increasing power densities (470 nm; 27, 54, 79, and 105 mWmm^−2^). *p* > 0.05; Kruskal–Wallis/Dunn’s tests (**d**, **e**); two-way ANOVA/Bonferroni’s tests (**f**); (Vm: *n* = 46, 36, and 36; Cm: *n* = 14, 15, and 16; Cm changes: 12–14, 14–15, and 16 for Ctrl, MTP2 5 µM and MTP2 10 µM, respectively

Next, we investigated the possible membrane potential modulation upon irradiation in HEK293T cells loaded with different concentrations of MTP2 (5 and 10 µM in saline) through the patch-clamp technique in whole-cell current-clamp configuration. Cells were stimulated with 20 ms light pulses at increasing power densities (27, 54, 79, and 105 mWmm^−2^) (Fig. 3). As shown in Fig. 3a, MTP2 photoisomerization induces a rapid depolarization of the membrane potential (3.0 ± 0.4 mV for 10 µM at 105 mWmm^−2^; Figs. 3b and S10) that reaches the peak within 10 ms after the onset of the light stimulus. Right after the end of the light stimulation, a fast repolarization of the membrane potential occurs, followed by a slight hyperpolarization (−0.7 ± 0.1 mV for 10 µM at 105 mWmm^−2^; Fig. 3c). The depolarization and hyperpolarization amplitudes are found to be dependent on both the MTP2 concentration and the light power density.

No significant changes in the resting membrane potential and capacitance were observed in HEK293T cells loaded with MTP2 (Fig. 3d, e). Furthermore, membrane capacitance was recorded while exciting the molecule with 200 ms light pulses. Interestingly, illumination does not result in significant variations in the membrane capacitance compared to the dark condition (Fig. 3f). This finding significantly differs from the insertion of Ziapin2 into the membrane, which induces an increase in cell capacitance, attributed to membrane thinning due to the formation of *trans*-Ziapin2 dimers across the membrane.

To further confirm the hypothesis that the partitioning of MTP2 does not induce any structural or mechanical distortion of the membrane, we tested the MTP2 effects in cells exogenously expressing mechano-sensitive channels (Fig. S11). For this experiment, the mechano-sensitive K^+^ channel TRAAK was chosen, as it has previously been shown to be activated by Ziapin2 insertion into the membrane^16^. Data revealed that, in HEK293T cells transfected with TRAAK, MTP2 generates a light-dependent depolarization accompanied by a photocurrent, whose amplitudes were comparable to those observed in non-transfected cells. These results dismiss the inference of MTP2 as a photo-mechanical modulator of the membrane thickness.

We also comparatively examined the light-induced physiological effects of the MTs family including MTA2, MTP1 and MTA1 (Fig. S12). When HEK293T cells were incubated in the presence of 10 µM of either compound, the light-evoked effects were qualitatively similar to those described for MTP2 and their magnitude progressively increased with the power density of the light stimulation (from 27 to 105 mW/mm^2^). However, the comparison clearly shows the superior performance of MTP2.

**Fig. 4:**
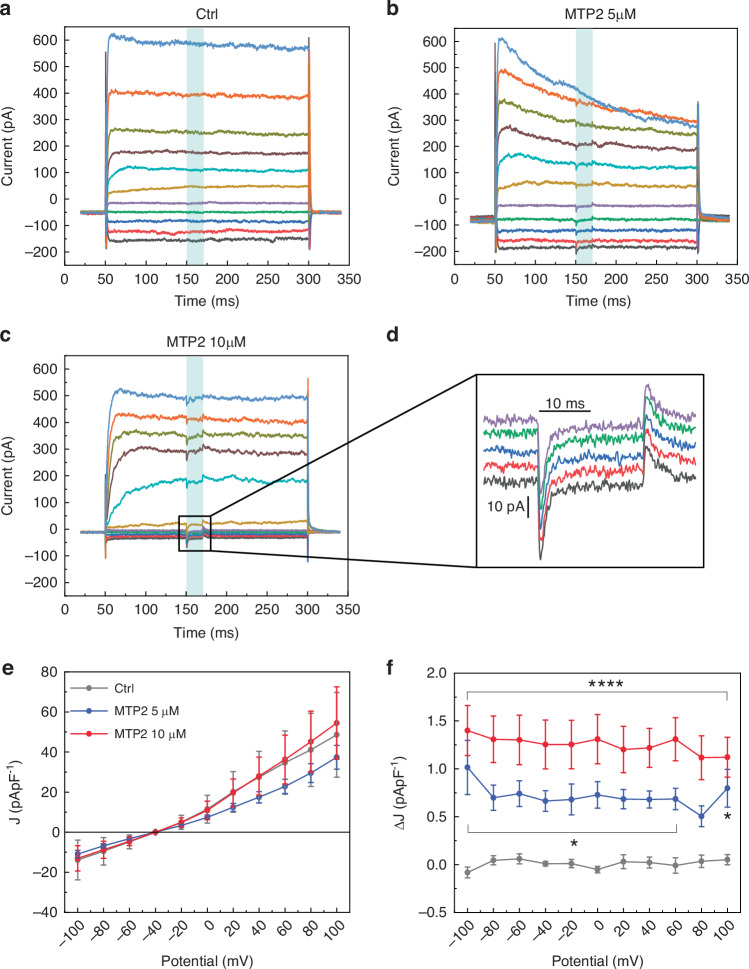
MTP2 induces an inward current in HEK293T cells upon light stimulation. Representative whole cell voltage clamp traces recorded from HEK293T cells incubated for 5 min in the presence of either vehicle (Ctrl; **a**), 5 µM MTP2 (**b**) or 10 µM MTP2 (**c**) and illuminated for 20 ms at 105 mWmm^−2^. The voltage step protocol is from –100mV to 100 mV with a step of 20 mV. **d** Higher magnification of voltage step protocol of 10 µM MTP2 treated cells ranging from –100mV to –20mV. Traces were zeroed for presentation purposes. **e**, **f** Graphs representing the current peak under dark conditions (**e**) and during illumination (**f**) under the same experimental conditions. Data are shown as mean ± s.e.m. *p < 0.05, ****p < 0.0001; two-way ANOVA/Bonferroni’s tests versus Ctrl. (*n* = 16, 12, and 15 for Ctrl, MTP2 5 µM and MTP2 10 µM, respectively)

Light-dependent response in cells loaded with MTP2 was also evaluated in the voltage-clamp configuration. Photocurrents are evoked by applying a short light pulse while maintaining cells at fixed potentials from –100 to 100 mV (Fig. 4). MTP2 photoisomerization generated a fast photocurrent that reached its maximum peak after about 2 ms; then, the photocurrent amplitude decayed maintaining a plateau for the entire illumination period (Fig. 4a–d). Under dark conditions, either in the presence or absence of MTP2, cells do not show any difference in current amplitude as a function of voltage. On the contrary, the light-evoked current depends on MTP2 concentration (Fig. 4e, f).

### Light-evoked modulation of the membrane potential in excitable cells: primary neurons and hiPSC-derived cardiomyocytes

Given the ability of MTP2 to modulate membrane potential upon light stimulation, an exploration of its effects on intrinsically excitable cells became imperative. To extend our understanding, the impact of the molecule was first evaluated on primary cultures of mouse hippocampal neurons. Initial assessments of biocompatibility revealed no significant differences in propidium iodide staining between control neurons and those incubated with various compounds at a concentration of 5 µM and specifically MTP2 also at 10 µM (Figs. S13 and 14).

**Fig. 5:**
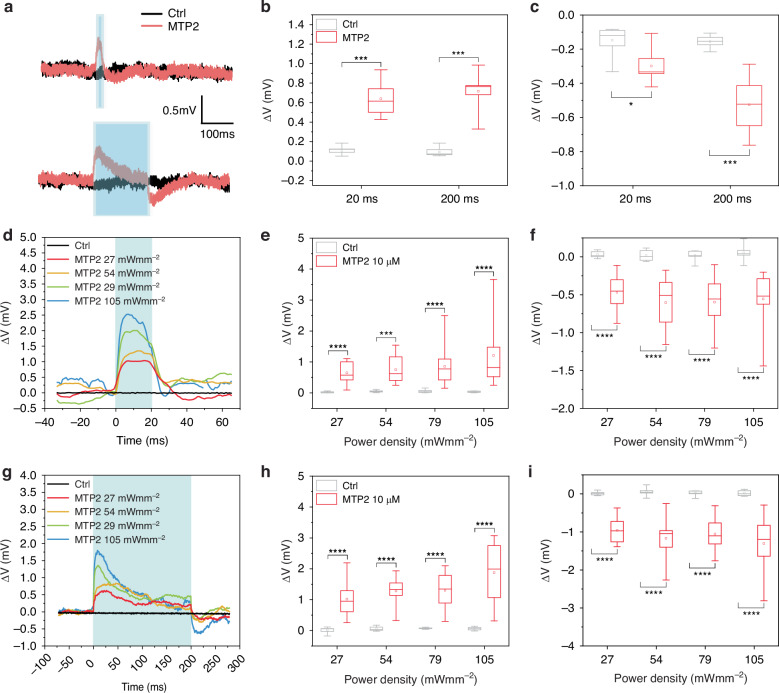
Light-evoked membrane voltage modulation by MTP2 in primary hippocampal neurons and hiPSC-CMs. **a** Representative whole-cell current clamp traces were recorded from primary hippocampal neurons incubated for 5 min in the presence of either vehicle (Ctrl, black) or 5 µM MTP2 (red) and illuminated for 20 ms (top) or 200 ms (bottom) at 20 mWmm^−2^. **b**, **c** Box plots of both peak depolarization (**b**) and hyperpolarization (**c**) in primary hippocampal neurons incubated for 5 min in the presence of either vehicle (Ctrl, grey boxes) or 5 µM MTP2 (red boxes) and illuminated for 20 or 200 ms at 20 mWmm^−2^. *p > 0.05; ***p < 0.001; Mann–Withney *U* test (**c**: *n* = 9 and 6 for Ctrl and MTP2 5 µM, respectively). **d**, **g** Representative whole-cell current clamp traces recorded from hiPSC-CMs incubated for 5 min in the presence of either vehicle (Ctrl, black) or 10 µM MTP2 (color) and illuminated for 20 ms (**d**) or 200 ms (**g**) at increasing power densities (27, 54, 79, and 105 mWmm^−2^). Traces were zeroed for presentation purposes. **e**, **f**, **h**, **i** Box plots of peak depolarization (**e**, **h**) and peak hyperpolarization (**f**, **i**) in hiPSC-CMs incubated for 5 min in the presence of either vehicle (Ctrl, grey boxes) or 10 µM MTP2 (red boxes). The experiments were performed at 35 °C. **p < 0.001; ****p < 0.0001; Student *t* test/Mann–Withney *U* test (**e**: *n* = 10 and 8–20; **f**: *n* = 8–10 and 8–19; **h**: *n* = 9–10 and 8; **i**: *n* = 9–10 and 8; for Ctrl and MTP2 10 µM, respectively)

Subsequently, patch-clamp experiments in whole-cell configuration were conducted on primary neurons loaded with the different molecules. Cells were stimulated with 20 and 200 ms light pulses in the cyan region of the spectrum (470 nm), and membrane voltage modulation was recorded within a 300 ms time window from light onset. The representative traces (Figs. 5a, b and S15A) illustrate that all molecules induced a transient depolarization. Notably, compounds featuring two polar branches (MTA2, MTP2) led to an approximate 1 mV depolarization, while those with a single branch (MTA1, MTP1) induced an average depolarization of 1.5 mV (Figs. 5c and S15B). Notably, the depolarization reached its maximum value within 20 ms after the light pulse onset. This phenomenon occurred even if the illumination was prolonged (200 ms). As shown in Fig. 5a, the depolarization returned to physiological values before the end of the stimulus likely due to the rise of intrinsic compensatory mechanisms. Although no differences in the amplitude of the evoked depolarization were observed between the two stimulation times, a rebound hyperpolarization became evident after the 200 ms stimulation, likely attributed to the long-lasting depolarization during light exposure. This data reaffirms the MTs’ ability to modulate membrane potential, extending our findings from HEK293T cells to primary neurons, even at the lowest concentration tested.

To further corroborate the functional impact of MTP2, we investigated its effectiveness in a distinct category of electrically excitable cells, specifically human-induced pluripotent stem cell-derived cardiomyocytes (hiPSC-CMs). To this purpose, we exposed hiPSC-CMs to 10 µM MTP2 and applied light stimulation, mirroring the protocols used for HEK293T and neuronal cells.

We verified the biocompatibility of hiPSC-CMs after the exposure of 10 µM MTP2 through the AlamarBlue assay. We monitored the cells between 4 and 72 h after MTP2 incubation, without detecting any significant differences in the AlamarBlue fluorescence, in agreement with the results obtained in both HEK293T cells and hippocampal neurons (Fig. S14).

Consistently with prior experimental findings, the photostimulation of MTP2-loaded hiPSC-CMs, using either short (20 ms, Fig. 5d–f) or long (200 ms, Fig. 5g–i) visible light pulses, induced a rapid depolarization that occurred immediately upon light onset. This depolarization gradually returned to physiological resting values, followed by a mild rebound hyperpolarization phase after the cessation of light. Notably, although the magnitude of the light-induced membrane potential modulation was less pronounced, the depolarization/hyperpolarization amplitudes exhibited an increasing trend, albeit not statistically significant, in response to higher light power densities, as observed in HEK293T cells.

### On the mechanism of membrane potential modulation by MT compounds

Molecular Dynamics simulations show that MTP2 has a strong affinity for the plasma membrane, and it enters the outer layer aligning with its main longitudinal axis with the acyl chains (Fig. 2). We hypothesize the transition of MTP2 between leaflets might occur in a sub-ms time scale via the so-called flip-flop mechanism. Such trans–bilayer motion of lipids, proteins and other molecules—which results in a mirror-image change in orientation with respect to the middle of the bilayer—is continuously happening in the plasma membrane, spontaneously or due to the action of specific membrane proteins and is also part of the membrane crossing process^25^. Although ions and charged molecules are generally unable to traverse cell membranes by themselves, relevant exceptions occur^26^, including cell penetrating peptides^27^. A general mechanism for charge translocation remains to be identified, but progress has been made for specific systems. For example, it has been shown that cations and positively charged compounds can permeate the membrane by inducing defects in the phospholipid layers as a result of strong electrostatic interactions with the headgroups^28^. Peptides bearing multiple charges cross via an intermediate step where they bridge defects in the opposite phospholipid layers, which are reduced when the charges are spaced apart^29^. In addition, it is known that the transmembrane voltage drives charge translocation^30^.

**Fig. 6:**
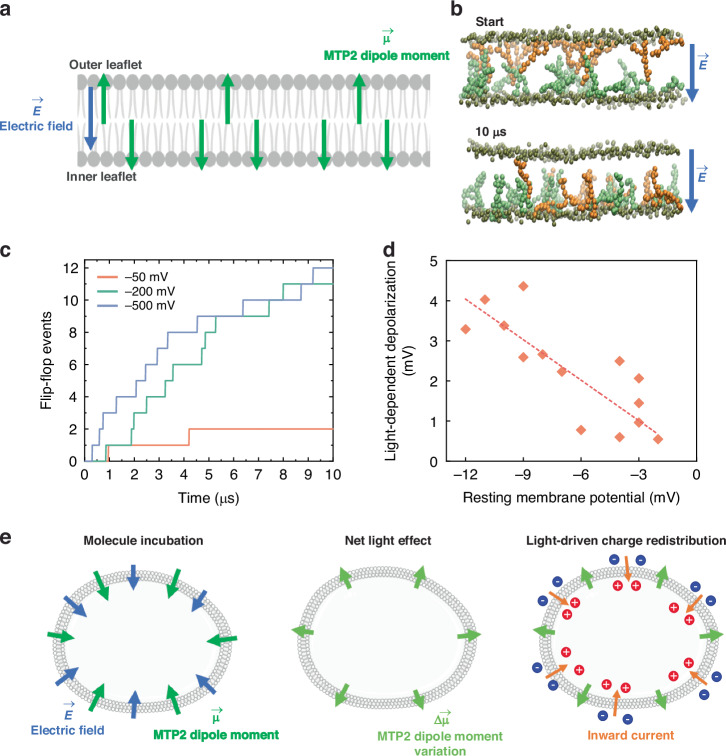
Proposed mechanism of light-driven cellular depolarization. **a** Sketch of the membrane lipid bilayer showing the preferential localization of the MTP2 molecules in the inner leaflet, as the molecular dipole moment tends to align to the electric field. **b** Snapshots of the system simulated with a voltage of −200 mV, starting (top) and final (bottom) conformation; orange and green MTP2 molecules represent the molecules bounded to the outer and inner leaflet at *t* = 0, respectively; water and lipid acyl chains are not reported for clarity. **c** Number of molecules moving from the outer to the inner leaflet during CG-MD simulations at different transmembrane voltages. **d** Plot showing the amplitude of the light-induced membrane depolarization as a function of the resting membrane potential in HEK293T cells, loaded with 10 μM MTP2 and stimulated with 20 ms 470 nm-light pulses at 105 mWmm^−2^. The dashed line represents the linear regression curve of the plotted data points (R^2^ = 0.88). **e** Sketch illustrating the MTP2 mechanism of light-driven membrane potential modulation in cells. The molecule is internalized within the membrane (left). When light is shined on the cells, an additional dipole moment appears (centre), which induces a redistribution of charges and an inward current across the membrane (right)

A confirmation of the uneven distribution of MTP2 in the two membrane leaflets comes from additional MD simulations. We performed CG-MD simulations in the presence of electric fields of different magnitudes, corresponding to −50, −200, and −500 mV transmembrane potentials^31^. We exploited a system comprising randomly distributed MTP2 molecules, 12 in the outer layer and 18 in the inner one. Results show that the higher the voltage, the more MTP2s flip from the outer to the inner leaflet, i.e. MTP2s dipole moments align with the direction of the electric field (Fig. 6a–c). All transition events occur in the µs range (Fig. 6c) and, in most of them, the molecule adopts a short-lived intermediate conformation with the two pyridine rings attached to the opposing layers (Fig. S18), in agreement with what is predicted for permeating systems with two positive charges^29^.

To estimate how MTP2 molecules partition between membrane layers, we followed the following reasoning, assuming that the driving force that determines the molecule orientation is the interaction of the intramembrane electric field with molecular dipole moment. By employing density-functional theory, we computed the permanent electrical dipole of MTP2 in both the *trans* and *cis* states (Fig. S17). In the *trans* state, MTP2 exhibits a dipole moment of 12.4 D, which decreases to 8.6 D upon photoisomerization to the *cis* form. Consequently, we expect that the preferential orientation of MTP2 will minimize the electrostatic energy, $U=\vec{\mu }\cdot \vec{E}$, as illustrated in Fig. 6a, with an energy difference $\varDelta U=2\mu E$ between the parallel and the antiparallel configuration. According to Boltzmann statistics, the ratio between the number of molecules whose dipole moment is parallel to the electric field and the ones which are antiparallel to the electric field is given by:

1 $$\frac{{N}_{{parallel}}}{{N}_{{antiparallel}}}=\exp \left(\frac{2\mu E}{{k}_{B}T}\right)$$

Plugging in the numerical values for the electrical dipole and the membrane electrical field (*E* ≈ 1 × 10^7^ Vm^−1^)^32^, we find ${N}_{{parallel}}\,/{N}_{{antiparallel}}=1.25$ at room temperature. There is thus a 25% excess fraction of MTP2 molecules that can induce a photostimulation effect due to a change in total dipole moment. This assumption is validated by the following experimental observation: there is a correlation between resting membrane potential and amplitude of the photoinduced depolarization (Fig. 6d). If we assume that the relation between the electric field and the resting membrane potential is linear, the higher the membrane potential, the higher the voltage drop across the membrane, thus, the higher the electric field. Accordingly, the observed correlation corroborates our assumptions. This finding has two main implications: (i) MTP2 molecules preferentially localize in the inner leaflet of the lipid bilayer as suggested by the Boltzmann relation in Eq. 1; (ii) the photoinduced modulation of the membrane potential observed in the cell is linked to the molecule dipole moment and its variation upon light excitation.

Shining light on the cells treated with the push–pull dye causes a reduction of the effective molecular dipole moment. Thus, the net differential light effect manifests as a dipole moment $\Delta \vec{\mu }$ in the membrane, pointing outward. For each MTP2 molecule undergoing isomerization, $\varDelta \mu \,=\,{\mu }_{{trans}}-{\mu }_{{cis}}$ = 3.8 *D* = 1.25 × 10^−29^ Cm. The emergence of $\Delta \vec{\mu }$ induces a rearrangement of the charges in the neighbourhood, resulting in the accumulation of positive charges at the inner leaflet of the membrane and negative charges at the outer leaflet (Fig. 6e). This charge displacement corresponds to the movement of ions across the membrane, which is consistent with the inward currents measured in the voltage-clamp experiments in Fig. 4. The ion readjustment leads to a change in the surface voltage across the membrane. After the 20 ms light pulse, MTP2 relaxes back to the *trans* isomer. However, within the duration of the light pulses, a dynamical equilibrium condition is reached: a constant number of push–pull azobenzenes are in the *cis* form as long as the cells remain illuminated. Upon switching off the light, the molecules gradually convert back to the *trans* conformation and the system relaxes to the original state, causing an opposite transient associated with hyperpolarization.

**Fig. 7:**
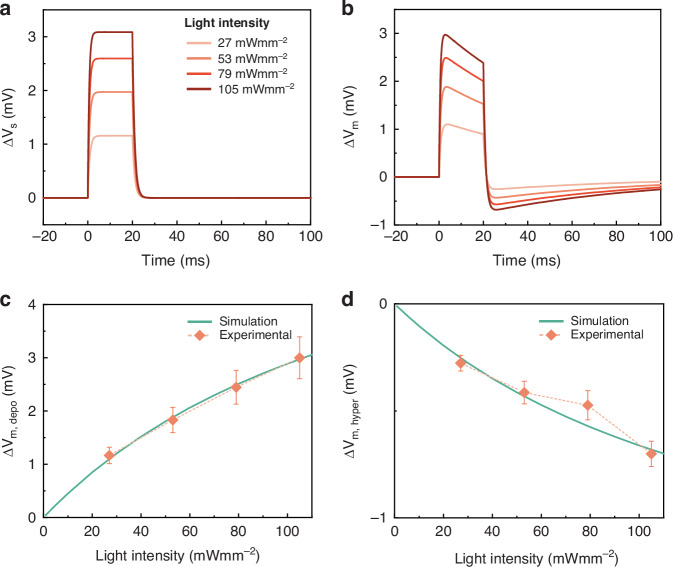
Mathematical model reproducing the temporal evolution of the membrane potential modulation by MTP2. Computed temporal profiles of the variation of the surface potential ${V}_{s}$ (**a**) and the corresponding membrane potential modulation (**b**) at different light intensities. **c**, **d** Maximum cell depolarization (**c**) and hyperpolarization (**d**) as a function of the light intensity used for excitation of MTP2 molecules. Data are expressed as mean ± s.e.m

As a final step in unveiling the mechanism of cell optostimulation, we devised a mathematical model that connects the photoisomerization dynamics to the modulation of the membrane potential. As outlined in the Supplementary Text, we adopt a well-known equivalent RC circuit picture^33–35^. In this circuit, the battery ${V}_{s}$ represents the surface potential, that is related to the asymmetric adsorption of ions on the two membrane sides^36^. The dipole moment variation due to the interaction of MTP2 with light prompts a displacement of charges that, in turn, modulates ${V}_{s}$. Assuming the change in ${V}_{s}$ be directly proportional to the number of molecules in the cis conformation, we utilized population dynamics equations for trans and cis isomers of MTP2 to replicate the observed membrane potential modulation (Fig. 7). The model reproduces the experimental time course and amplitude of the depolarization and hyperpolarization signals at the various light intensities employed in the patch-clamp measurements.

## Discussion

In this work, we introduce a novel approach for non-genetic cell optostimulation, based on intramembrane light-responsive dipole moment modulators. Light absorption by push–pull azobenzenes dwelling in the membrane causes a change in the surface charge triggered by the molecular dipole electric field. This variation is associated with a displacement of charges due to a movement of ions across the membrane, coherent with experimentally measured inward currents. This phenomenon enlarges the range of the existing cell opto-stimulation mechanisms, traditionally based on opto-capacitance, electrostatic coupling, ion channel gating or membrane poration^37^. Here, the push–pull azobenzenes MTs, characterized by an electron donor group and a nitro electron withdrawing group, offer advantages over previously tested aminoazobenzenes that operate as optomechanical actuators. Notably, they exhibit high water solubility, higher trans–cis interconversion rate and, crucially, they remain inactive in the dark state. MTs have been designed to exhibit an amphiphilic character able to interact with the lipid bilayer, not unlike the reported photoswitchable lipids^38^. The main difference resides in the stimulation mechanism after the initial intermolecular interaction due to the amphiphilicity. Namely, here we present a new mechanism linked to other light-sensitive properties of the photoswitchable core, the molecular dipole moment.

Through a screening of a homologous series of compounds, mainly by cell association assay, MTP2 emerged as the most promising system, demonstrating enhanced affinity with the cell membrane. We thus focused on MTP2 and we assessed its push–pull nature and its interaction with the membrane environment using steady-state optical spectroscopy and MD simulations. Subsequent ultrafast time-resolved techniques allowed for the study of the evolution of the excited state, providing insights into the isomerization reaction. We then evaluated the light-evoked membrane potential modulation driven by MTP2 in cell lines, primary neurons and hiPSC-CMs, observing clear and reproducible depolarization peaks.

Finally, leveraging MD simulations and DFT calculations of the molecular dipole moment, we developed a mathematical model to unveil and reproduce the temporal evolution of the membrane potential upon photostimulation. The model is based on the molecular dipole moment variation between *trans* and *cis* states. Our model successfully replicates the membrane potential modulation induced by MTP2 at varying light intensities. Considering these features, MTP2 emerges as a non-genetic optostimulation tool, capitalizing on the precise modulation of relevant electric characteristics of the lipid membrane.

Remarkably, the depolarization amplitude was insufficient to initiate an action potential. Our mathematical model indicates that the photoresponse is mainly due to the asymmetric distribution of MTP2 in the leaflets; the inner leaflet contains 25% more molecules than the outer leaflet and we hypothesize that increasing this asymmetry could enhance the photoresponse amplitude. To boost light-dependent depolarization and enable MTP2 to trigger action potentials, azobenzene could be anchored to external scaffolds, such as nanoparticles, organic sheets or fibers, to force the molecules orientation in a specific direction.

However, the rapid light-induced depolarization, although insufficient to trigger an action potential, holds potentiality for other advanced applications. Specifically, we envision to explore MTP2-mediated sub-threshold optical stimulation to destabilize and terminate re-entry-based arrhythmias (e.g., spiral waves), an approach recently proved successful both in silico and in experimental model systems using optogenetics^39–41^. In conclusion, MTP2, capitalizing on the precise modulation of relevant electric characteristics of the lipid membrane, emerges as a non-genetic optostimulation tool with potential therapeutic applications in biomedicine and biophotonics.

## Materials and methods

### Synthesis and chemical characterization

Unless otherwise stated, all chemicals and solvents were commercially available and used without further purification. Thin-layer chromatography was performed using silica gel on aluminium foil (Sigma Aldrich); ^1^H and ^13^C NMR spectra were collected with a Bruker ARX400. Mass spectroscopy was carried out with a Bruker Esquire 3000 plus. For details about the synthesis see supplementary information.

### Steady-state UV–Vis and PL measurements

UV–Vis absorption measurements were performed employing a Varian Cary5000 spectrophotometer and a Perkin ElmerLambda 1050 spectrophotometer, with deuterium (180–320 nm) and tungsten (320–3300 nm) lamps, a monochromator and three detectors (photomultiplier 180–860 nm, InGaAs 860–1300 nm, and PbS 1300–3300 nm). Absorption spectra were normalized according to a reference spectrum taken at 100% transmission (without the sample), 0% transmission (with an internal shutter), and in the presence of the reference solvent. For the PL measurements, an iHR320Horiba NanoLog Fluorometer was used, equipped with a xenon lamp, two monochromators, and two detectors (photomultiplier and InGaAs).

### µs TA measurements

The probe pulse originates from an amplified femtosecond laser (Light Conversion Pharos) producing pulses of 280 fs centred at 1030 nm. A broadband white light probe is generated by focusing the light beam into a 1 mm sapphire plate. The pump 1 ns–long pulses at 532 nm are provided by the second harmonic of a Q-switched Nd:YVO4 laser (Innolas Picolo), which was electronically triggered and synchronized to the femtosecond laser via an electronic delay generator. Experiments were performed with a repetition rate of 2 Hz and an average pump power of 3.5 mW. The detection system is like the one described above. The fittings of the temporal dynamics are performed by exploiting the particle swarm optimization MATLAB algorithm, using a monoexponential model. I also implemented a custom MATLAB function to consider the fact that, in some cases, the dynamics are slow and there is a pile-up effect.

### Ultrafast TA measurements

TA experiments exploited as a light source a regeneratively amplified Ti:sapphire system (Coherent Libra II), emitting 100 fs pulses centred at 800 nm at 1 kHz repetition rate with 4 W average power. The pump pulses were generated by exploiting a frequency-tuneable noncollinear optical parametric amplifier (NOPA)^42^ that can produce 100 fs narrow band pulses at all the wavelengths from 480 nm to 1.6 μm. For the experiments described in this work, the NOPA was pumped at 400 nm by the second harmonic of the laser, which is generated in a 2-mm thick beta-barium borate (BBO) crystal and is seeded by a white-light continuum (WLC), generated in a 1 mm-thick sapphire plate and then spectrally cut with an interferential filter centred at 500 nm with 10 nm bandwidth. The average pump power was set as 50 µW. The seed is then amplified in a 2 mm type-1 BBO crystal. To generate the broadband probe pulse, a small portion of the fundamental beam was focused into a 2 mm-thick sapphire crystal, producing a WLC. Filtering out the fundamental with BG39 filers, it is possible to obtain probe pulses in the visible range (420–730 nm). The pump-probe delay was controlled by a motorized delay stage and the transmitted probe signal was collected by an optical multichannel amplifier.

### Molecular dynamics simulations

The spontaneous insertion of *trans-*MTP2 in the membrane was studied by running 5 independent, 500 ns long all-atom MD simulations with one MTP2 molecule starting in water near a membrane bilayer of pure 1-palmitoyl-2-oleoylphosphatidylcholine (POPC, 180 molecules). To investigate the light-dependent effects of MTP2 on the membrane structure, we set up two simulation systems comprising eight MTP2 in trans or cis conformation within a bilayer of 180 POPC lipids. Four molecules per leaflet were distributed and the trajectories lasted 1 µs each. All systems were prepared using the CHARMM-GUI platform^43^, while the simulations were run with the NAMD v2.12 code^44^. The CHARMM36 force field^45^ and the TIP3P water model were used. CHARMM-compatible topology, parameters, and atomic charges for MTP2 were obtained from the CHARMM General force field^46^. A physiological concentration of counter ions was used to neutralize the total system charge. Simulations were run at constant pressure (1 atm) and temperature (310 K), i.e., in the NPT ensemble, using a Langevin piston^47^ with a constant decay of 100 ps^−^^1^ and an oscillation period of 200 fs, and a Langevin thermostat with a damping constant of 5 ps. Long-range electrostatic interactions were computed using the particle-mesh Ewald method^48^, with a fourth-order spline and 1 Å grid spacing. A time step of 2 fs was employed. The trajectories were analyzed using the MEMBPLUGIN tool^49^ and the VMD program^50^.

CG simulations were performed using the MARTINI v2.2 force field^51^. Based on the force field rule, the MTP2 atoms were mapped into 18 beads using the CG Builder tool^52^. Non-bonded interactions were derived from previously established Martini v2.2 models, while bonded parameters were determined by an iterative procedure based on comparison with the all-atom molecular features. The interaction strength of a few MTP2 CG beads with lipid molecules was tuned to reproduce the average orientation of MTP2 observed in the all-atom simulations, without affecting lipid-lipid interactions. A system comprising 120 *trans*-MTP2 molecules in a 270 × 270 Å^2^ bilayer of 1942 POPC lipids, water, and antifreeze molecules, was built using the Insane tool^53^. A solvated POPC membrane of the same size was also prepared as a control. CG-MD simulations were performed with the GROMACS simulation package (version 2022.3)^54^ and lasted 10 μs, using a time step of 20 fs. The temperature was maintained at 310 K via velocity rescaling (with characteristic time 1 ps)^55^, while the pressure was kept at 1 atm with the Berendsen semi-isotropic pressure coupling^56^. The membrane thickness was computed with the SuAVE software, which explicitly considers local bilayer curvature effects^57^.

Three additional CG-MD simulations were performed in presence of different values of transmembrane potential (–50 mV, –200 mV, –500 mV), generated by applying a constant external electric field $E=V/{L}_{z}$, where $V$ is the desired voltage value and ${L}_{z}$ is the length of the simulation box in the direction of the applied field^31^. Simulations were performed at constant volume and temperature (NVT ensemble) for a system of 30 *trans*-MTP2 molecules initially randomly displaced between layers. All other simulation settings and parameters were the same as the previous CG-MD trajectory.

### HEK293T cell cultures

In vitro electrophysiological experiments were performed using the immortalized cell line HEK293T (Human Embryonic Kidney), purchased from ATCC. HEK293T cells were cultured in T-25 cell culture flasks containing Dulbecco’s Modified Eagle Medium high glucose (DMEM-HG) culture medium, supplemented with 10% heat-inactivated Fetal Bovine Serum and 1% GlutaMAX (200 mM). Culture flasks were maintained in a humidified incubator at 37 °C with 5% CO_2_. When at 80% of confluence, cells were enzymatically detached from the flasks with a 1× trypsin-EDTA solution, plated on sterilized substrates and left to grow for 24 h before the transfection. Prior to cell plating, a layer of fibronectin (2 μgmL^−1^ in PBS buffer solution) was deposited on the sample surface and incubated for 1 h at 37 °C to promote cellular adhesion. To exogenously express TRAAK channels, cells were transfected with a pIRES: hTRAAK plasmid, purchased from Addgene. pIRES: hTRAAK was a gift from Dan Minor (Addgene plasmid #133080; http://n2t.net/addgene:133080; RRID:Addgene_133080)^58^. Transfection was performed using LipofectamineTM 3000 reagent (Life Technologies). Cells were then incubated with a cocktail of LipofectamineTM 3000 reagent and 1 ng of pIRES:hTRAAK purified plasmid for 5 h following the traditional procedures. Twenty four hours after the transfection, cells were ready for the electrophysiological experiments.

To preliminarily evaluate the molecule cytotoxicity, AlamarBlue proliferation assay was performed. For this experiment, 4000 cellscm^-^^2^ were plated one day before the experiment. The samples were incubated with different concentrations of MTP2 (5 and 10 μM) for 5 min. Next, half of the samples were exposed to light (470 nm, 200 μWmm^−2^, 30 s). The AlamarBlue Reagent (Invitrogen DAL 1100) was diluted 1:10 with DMEM without phenol red. The solution without the cell was used as blank. The samples were incubated for 4 h and subsequently transferred to a 96-well plate. The fluorescence was detected using a spectrophotometer, exciting at 560 nm and measuring the emission at 590 nm. The treatment with the AlamarBue reagent was repeated 24, 48, and 96 h after the incubation.

### Primary hippocampal neuron cultures

Primary hippocampal neurons were obtained from WT C57BL/6 J mice sourced from Charles River (Calco, Italy). Animals were anesthetized by CO_2_ inhalation and sacrificed by cervical dislocation. Embryos at embryonic day 17/18 were immediately extracted via caesarean section. Hippocampi were dissected in iced cold media and then incubated in 0.25% Trypsin-EDTA (Gibco ThermoFisher Scientific, Segrate, Italy) at 37 °C for 15 min for enzymatic digestion. Subsequently, the cells were mechanically dissociated using a fire-polished Pasteur pipette, and cell viability was assessed using the Trypan Blue exclusion assay. Neurons were plated onto poly-d-lysine (0.1 mg/ml, Sigma-Aldrich)-coated 18-mm glass coverslips (4 × 10^4^ cells/coverslip) in the culture media containing Neurobasal media (Gibco) supplemented with 2% B27 (Gibco), 0.5 mM Glutamate (Gibco), and 1% Penicillin–Streptomycin (Gibco) and kept at 37 °C in a 5% CO_2_ humidified atmosphere. All experiments were performed at 14–18 days in vitro.

For assessing neuronal viability, primary hippocampal neurons were either incubated with their vehicle or with MT molecules (5 µM) for 5 min. Cells were then stained with propidium iodide (PI; 1 µM) for cell death and with Hoechst 33342 (1 µM) for nuclei visualization for further 5 min. Images were acquired at 10× (0.5 NA) magnification using an Eclipse-80i upright epifluorescence microscope (Nikon Instruments). Cell viability was obtained by the ratio of Propidium Iodide (PI)-/Hoechst 33342-positive cells. Image analysis was performed using the ImageJ software and the Cell Counter plugin.

### hiPSCs culture and differentiation to hiPSC-CMs

The human induced pluripotent stem cell (hiPSC) line was obtained from the National Institute of General Medical Sciences Human Genetic Cell Repository at the Coriell Institute for Medical Research: GM25256. hiPSCs were cultured on recombinant human vitronectin (rhVTN, ThermoFisher) in E8 Flex medium (ThermoFisher) and differentiated towards hiPSC-derived cardiomyocytes (hiPSC-CMs) on cell culture-grade Matrigel (BD). Ventricular-like hiPSC-CMs were differentiated from hiPSCs by using a previously published protocol based on the modulation of the Wnt-signalling pathway^59^, purified through glucose starvation to obtain a population with more than 90% of CMs and cryopreserved in serum-free 10% DMSO-based cryopreservation medium before day 20. Cryopreserved hiPSC-CMs were thawed before each experiment and maintained in culture for two weeks in RPMI 1640 medium supplemented with 1× B27 Supplement and 1% KnockOut Serum Replacement (ThermoFisher) as previously described^60^. The medium was refreshed every other day. Beating monolayers of hiPSC-CMs were dissociated with TrypLE 1× (ThermoFisher) and seeded on 18 mm Ø glass coverslips pre-coated with cell culture-grade Matrigel (BD) at a density of 8.5 to 12.5 × 10^3^ cellscm^−2^. To assess the molecule cytotoxicity, AlamarBlue proliferation assay was performed as previously reported for HEK293T cell cultures. hiPSC-CMs were seeded at 1.5 × 10^5^ and 3 × 10^5^ cellscm^−2^ on Matrigel coated 96-well plates from Greiner.

### Patch-clamp electrophysiology

#### HEK293T cells

Standard patch clamp recordings were performed with an Axopatch 200B (Axon Instruments) coupled with a Nikon Eclipse Ti inverted microscope. HEK293T cells were measured in whole-cell configuration with freshly pulled glass pipettes (4–7 MΩ), filled with the following intracellular solution [mM]: 12 KCl, 125 K-Gluconate, 1 MgCl_2_, 0.1 CaCl_2_, 10 EGTA, 10 HEPES, and 10 ATP-Na_2_. The extracellular solution contained [mM] 135 NaCl, 5.4 KCl, 5 HEPES, 10 Glucose, 1.8 CaCl_2_, and 1 MgCl_2_. The acquisition was performed with pClamp-10 software (Axon Instruments). Membrane currents were low pass filtered at 2 kHz and digitized with a sampling rate of 10 kHz (Digidata 1440 A, Molecular Devices). Cell membrane capacitance (*C*_*m*_) was measured by applying a voltage step of 5 mV. The capacitance current area (ΔQ) was calculated using Origin software. *C*_*m*_ was calculated as *C*_*m*_ = ΔQ/ΔT.

#### Primary neurons

Whole-cell patch clamp recordings of low-density and autaptic primary hippocampal neurons were performed in the dark at room temperature (22–24 °C) using an EPC10 (HEKA Elektronik, Reutlingen, Germany) amplifier and the PatchMaster program (HEKA). Borosilicate glass pipettes (Kimble, Kimax, Mexico) with a 3–4 MΩ resistance were used. Pipettes were filled with an intracellular solution containing [mM]: 126 K gluconate, 4 NaCl, 1 MgSO_4_, 0.02 CaCl_2_, 0.1 BAPTA, 15 glucose, 5 HEPES, 3 ATP, and 0.1 GTP (pH 7.3 with KOH). Cells were maintained in standard extracellular Tyrode solution containing (mM]: 140 NaCl, 2 CaCl_2_, 1 MgCl_2_, 4 KCl, 10 glucose, and 10 HEPES (pH 7.3 with NaOH). All chemicals were purchased from Sigma Aldrich (St. Louis, MO, USA). Cells with leak currents >100 pA or series resistance (R) > 15 Ω were discarded. Hippocampal neurons were incubated with the vehicle or MTs (5 µM) for 5 min at room temperature and then washed twice with the extracellular medium. Data analysis employed FitMaster software version 2.71 (HEKA) and Origin 8.6 (OriginLab Corporation; Northampton). To study light-dependent membrane voltage modulation in low-density neurons synaptically isolated from the neuronal network, D-AP5 (50 µM), CNQX (10 µM) and Bicuculline (30 µM) were added to the extracellular medium to block excitatory and inhibitory transmissions. Current-clamp recordings of light-induced depolarization were performed by holding the cell at its resting membrane potential (Ih = 0) and stimulating it with 7 subsequent 20 ms or 200 ms light pulses. The resulting depolarization was calculated in a 300 ms time window after each light pulse and compared to the basal membrane potential before stimulation.

#### hiPSC-CMs

The cells underwent perfusion with an extracellular solution composed of [mM]: 154 NaCl, 4 KCl, 5 HEPES NaOH, 2 CaCl_2_, 1 MgCl_2_, 5.5 glucose (pH 7.35 with NaOH). Patch pipettes with a resistance of 1.5–2.5 MΩ were filled with an intracellular solution containing [mM]: 110 K-aspartate, 23 KCl, 3 MgCl_2_, 0.04 CaCl_2_, 0.1 EGTA KOH (10^−7^ Ca^2+^-free), 5 HEPES KOH, 0.4 Na^+^-GTP, 5 Na^+^-ATP, 5 Na^+^-phosphocreatine (pH 7.3 with KOH). The intracellular solution was cryopreserved at −20 °C and thawed before each experiment. Membrane capacitance and series resistance (<10 MΩ) were measured in every cell but left uncompensated. Signals were amplified using Axopatch 200B (Axon Instruments), digitized at 10 kHz (Axon Digidata 1440 A, Molecular Devices), and filtered at 2 kHz. Following a 5-min incubation at room temperature with either the vehicle or MTP2 (10 µM), hiPSC-CMs were washed twice with the extracellular medium. All experiments were carried out at a physiological temperature (35 °C).

#### Photostimulation

Illumination of cells during electrophysiological experiments was provided by an LED system (Lumencor Spectra X) coupled to the fluorescence port of the microscope and characterized by a maximum emission wavelength of 474 nm to match the molecule absorption spectrum. For HEK293T cells and hiPSC-CMs the illuminated spot on the sample has an area of 0.23 mm^2^ and a photoexcitation density of 27, 54, 79, and 105 mWmm^−2^, while for primary neurons the power density was 20 mWmm^−2^, as measured at the output of the microscope objective.

### Statistical analysis

Data are all expressed as box plots. The box plot elements are the following: centre line, median (Q2); cross symbol, mean; box limits, 25^th^ (Q1)–75^th^ (Q3) percentiles; whisker length is determined by the minimum and the maximum value. Normal distribution was assessed using D’Agostino and Pearson’s normality test. To compare two samples, either the Student’s *t* test or the Mann–Whitney’s *U* test was used, depending on the normality. To compare more than two samples, either one-way ANOVA or the Kruskal–Wallis test was used followed by either Bonferroni’s, Holm-Sidak’s, Dunnett’s or Dunn’s multiple comparison test. For multiple variables or repeated measures, a two-way ANOVA test was used. The significance level was preset to *p* < 0.05 for all tests. Statistical analysis was carried out using GraphPad Prism 6 software.

## Supplementary information

Supplementary Material

## Data Availability

The data and material that support the findings of this study are available upon request to the corresponding authors.

## Materials availability

The material that supports the findings of this study are available upon request to the corresponding authors.

## References

[1] Fenno, L., Yizhar, O. & Deisseroth, K. The development and application of optogenetics. *Annu. Rev. Neurosci.* **34**, 389–412 (2011). 21692661 10.1146/annurev-neuro-061010-113817 PMC6699620

[2] Scanziani, M. & Häusser, M. Electrophysiology in the age of light. *Nature* **461**, 930–939 (2009). 19829373 10.1038/nature08540

[3] Thompson, A. C., Stoddart, P. R. & Jansen, E. D. Optical stimulation of neurons. *Curr. Mol. Imaging* **3**, 162–177 (2015). 10.2174/2211555203666141117220611 PMC4541079 26322269

[4] Di Maria, F. et al. The evolution of artificial light actuators in living systems: from planar to nanostructured interfaces. *Chem. Soc. Rev.* **47**, 4757–4780 (2018). 29663003 10.1039/c7cs00860k

[5] Rau, H. Azo compounds. In *Photochromism: Molecules and Systems* (eds Dürr, H. & Bouas-Laurent, H.) 165–192 (Amsterdam: Elsevier, 2003).

[6] Beharry, A. A. & Woolley, G. A. Azobenzene photoswitches for biomolecules. *Chem. Soc. Rev.* **40**, 4422–4437 (2011). 21483974 10.1039/c1cs15023e

[7] Tochitsky, I. et al. Restoring vision to the blind with chemical photoswitches. *Chem. Rev.* **118**, 10748–10773 (2018). 29874052 10.1021/acs.chemrev.7b00723 PMC6389275

[8] Szymański, W. et al. Reversible photocontrol of biological systems by the incorporation of molecular photoswitches. *Chem. Rev.* **113**, 6114–6178 (2013). 23614556 10.1021/cr300179f

[9] Bregestovski, P., Maleeva, G. & Gorostiza, P. Light-induced regulation of ligand-gated channel activity. *Br. J. Pharmacol.* **175**, 1892–1902 (2018). 28859250 10.1111/bph.14022 PMC5979632

[10] DiFrancesco, M. L. et al. Neuronal firing modulation by a membrane-targeted photoswitch. *Nat. Nanotechnol.* **15**, 296–306 (2020). 32015505 10.1038/s41565-019-0632-6

[11] Paternò, G. M. et al. The effect of an intramembrane light-actuator on the dynamics of phospholipids in model membranes and intact cells. *Langmuir* **36**, 11517–11527 (2020). 32903010 10.1021/acs.langmuir.0c01846

[12] Magni, A. et al. Azobenzene photoisomerization probes cell membrane viscosity. *Phys. Chem. Chem. Phys.* **24**, 8716–8723 (2022). 35373231 10.1039/d1cp05881a

[13] Paternò, G. M. et al. Membrane environment enables ultrafast isomerization of amphiphilic azobenzene. *Adv. Sci.* **7**, 1903241 (2020). 10.1002/advs.201903241 PMC7175258 32328424

[14] Venturino, I. et al. Skeletal muscle cells opto-stimulation by intramembrane molecular transducers. *Commun. Biol.* **6**, 1148 (2023). 37952040 10.1038/s42003-023-05538-y PMC10640616

[15] Vurro, V. et al. Optical modulation of excitation-contraction coupling in human-induced pluripotent stem cell-derived cardiomyocytes. *iScience* **26**, 106121 (2023). 36879812 10.1016/j.isci.2023.106121 PMC9984557

[16] de Souza-Guerreiro, T. C. et al. Membrane targeted azobenzene drives optical modulation of bacterial membrane potential. *Adv. Sci.* **10**, 2205007 (2023). 10.1002/advs.202205007 PMC10015841 36710255

[17] Moschetta, M. et al. Modulation of mechanosensitive potassium channels by a membrane-targeted nongenetic photoswitch. *J. Phys. Chem. B* **127**, 8869–8878 (2023). 37815392 10.1021/acs.jpcb.3c04551 PMC10591468

[18] Martinac, B., Adler, J. & Kung, C. Mechanosensitive ion channels of *E*. *coli* activated by amphipaths. *Nature* **348**, 261–263 (1990). 1700306 10.1038/348261a0

[19] Bassetto, C. A. Z. et al. Photolipid excitation triggers depolarizing optocapacitive currents and action potentials. *Nat. Commun.* **15**, 1139 (2024). 38326372 10.1038/s41467-024-45403-y PMC10850502

[20] Vurro, V. et al. Molecular design of amphiphilic plasma membrane-targeted azobenzenes for nongenetic optical stimulation. *Front. Mater.* **7**, 631567 (2021).

[21] Bandara, H. M. D. & Burdette, S. C. Photoisomerization in different classes of azobenzene. *Chem. Soc. Rev.* **41**, 1809–1825 (2012). 22008710 10.1039/c1cs15179g

[22] Tulumello, D. V. & Deber, C. M. SDS micelles as a membrane-mimetic environment for transmembrane segments. *Biochemistry* **48**, 12096–12103 (2009). 19921933 10.1021/bi9013819

[23] Lakowicz, J. R. *Principles of Fluorescence Spectroscopy.* 3rd edn. (New York: Springer, 2006).

[24] Gille, K., Knoll, H. & Quitzsch, K. Rate constants of the thermal cis-trans isomerization of azobenzene dyes in solvents, acetone/water mixtures, and in microheterogeneous surfactant solutions. *Int. J. Chem. Kinet.* **31**, 337–350 (1999).

[25] Parisio, G., Ferrarini, A. & Sperotto, M. M. Model studies of lipid flip-flop in membranes. *Int. J. Adv. Eng. Sci. Appl. Math.* **8**, 134–146 (2016).

[26] Deamer, D. W. & Bramhall, J. Permeability of lipid bilayers to water and ionic solutes. *Chem. Phys. Lipids* **40**, 167–188 (1986). 2427233 10.1016/0009-3084(86)90069-1

[27] Wagstaff, K. M. & Jans, D. A. Protein transduction: cell penetrating peptides and their therapeutic applications. *Curr. Med. Chem.* **13**, 1371–1387 (2006). 16719783 10.2174/092986706776872871

[28] Vorobyov, I. et al. Ion-induced defect permeation of lipid membranes. *Biophys. J.* **106**, 586–597 (2014). 24507599 10.1016/j.bpj.2013.12.027 PMC3945052

[29] Povilaitis, S. C. et al. Design of peptides for membrane insertion: the critical role of charge separation. *J. Phys. Chem. B* **126**, 6454–6463 (2022). 35997537 10.1021/acs.jpcb.2c04615 PMC9541189

[30] Rothbard, J. B., Jessop, T. C. & Wender, P. A. Adaptive translocation: the role of hydrogen bonding and membrane potential in the uptake of guanidinium-rich transporters into cells. *Adv. Drug Deliv. Rev.* **57**, 495–504 (2005). 15722160 10.1016/j.addr.2004.10.003

[31] Roux, B. The membrane potential and its representation by a constant electric field in computer simulations. *Biophys. J.* **95**, 4205–4216 (2008). 18641071 10.1529/biophysj.108.136499 PMC2567939

[32] Stroud, R. M. et al. Glycerol facilitator GlpF and the associated aquaporin family of channels. *Curr. Opin. Struct. Biol.* **13**, 424–431 (2003). 12948772 10.1016/s0959-440x(03)00114-3

[33] Pinto, B. I., Bassetto, C. A. Z. & Bezanilla, F. Optocapacitance: physical basis and its application. *Biophys. Rev.* **14**, 569–577 (2022). 35528029 10.1007/s12551-022-00943-9 PMC9042976

[34] Vurro, V. et al. Photostimulation mechanism of an amphiphilic azobenzene. *IL Nuovo Cimento C* **46**, 143 (2023).

[35] Magni, A., Vurro, V. & Lanzani, G. The effects of reiterated cell photo-stimulation with an azobenzene. *IL Nuovo Cimento C* **46**, 154 (2023).

[36] Plaksin, M. et al. Thermal transients excite neurons through universal intramembrane mechanoelectrical effects. *Phys. Rev. X* **8**, 011043 (2018).

[37] Magni, A. et al. A membrane intercalating metal-free conjugated organic photosensitizer for bacterial photodynamic inactivation. *Chem. Sci.* **14**, 8196–8205 (2023). 37538813 10.1039/d3sc01168b PMC10395270

[38] Morstein, J., Impastato, A. C. & Trauner, D. Photoswitchable lipids. *ChemBioChem* **22**, 73–83 (2021). 32790211 10.1002/cbic.202000449

[39] Hussaini, S. et al. Drift and termination of spiral waves in optogenetically modified cardiac tissue at sub-threshold illumination. *eLife* **10**, e59954 (2021). 33502313 10.7554/eLife.59954 PMC7840178

[40] Biasci, V. et al. Optogenetic manipulation of cardiac electrical dynamics using sub-threshold illumination: dissecting the role of cardiac alternans in terminating rapid rhythms. *Basic Res. Cardiol.* **117**, 25 (2022). 35488105 10.1007/s00395-022-00933-8 PMC9054908

[41] Marchal, G. A. et al. Optogenetic manipulation of cardiac repolarization gradients using sub-threshold illumination. *Front. Physiol.* **14**, 1167524 (2023). 37215182 10.3389/fphys.2023.1167524 PMC10196067

[42] Manzoni, C. & Cerullo, G. Design criteria for ultrafast optical parametric amplifiers. *J. Opt.* **18**, 103501 (2016).

[43] Jo, S. et al. CHARMM-GUI: a web-based graphical user interface for CHARMM. *J. Comput. Chem.* **29**, 1859–1865 (2008). 18351591 10.1002/jcc.20945

[44] Phillips, J. C. et al. Scalable molecular dynamics with NAMD. *J. Comput. Chem.* **26**, 1781–1802 (2005). 16222654 10.1002/jcc.20289 PMC2486339

[45] Huang, J. & Mackerell, A. D. Jr CHARMM36 all-atom additive protein force field: validation based on comparison to NMR data. *J. Comput. Chem.* **34**, 2135–2145 (2013). 23832629 10.1002/jcc.23354 PMC3800559

[46] Vanommeslaeghe, K. et al. CHARMM general force field: a force field for drug-like molecules compatible with the CHARMM all-atom additive biological force fields. *J. Comput. Chem.* **31**, 671–690 (2010). 19575467 10.1002/jcc.21367 PMC2888302

[47] Feller, S. E. et al. Constant pressure molecular dynamics simulation: the Langevin piston method. *J. Chem. Phys.* **103**, 4613–4621 (1995).

[48] Darden, T., York, D. & Pedersen, L. Particle mesh Ewald: an *N*⋅log(*N*) method for Ewald sums in large systems. *J. Chem. Phys.* **98**, 10089–10092 (1993).

[49] Guixà-González, R. et al. MEMBPLUGIN: studying membrane complexity in VMD. *Bioinformatics* **30**, 1478–1480 (2014). 24451625 10.1093/bioinformatics/btu037

[50] Humphrey, W., Dalke, A. & Schulten, K. VMD: visual molecular dynamics. *J. Mol. Graph.* **14**, 33–38 (1996). 8744570 10.1016/0263-7855(96)00018-5

[51] de Jong, D. H. et al. Improved parameters for the martini coarse-grained protein force field. *J. Chem. Theory Comput.* **9**, 687–697 (2013). 26589065 10.1021/ct300646g

[52] Barnoud, J. CG builder. available on line https://jbarnoud.github.io/cgbuilder/.

[53] Wassenaar, T. A. et al. Computational lipidomics with *insane*: a versatile tool for generating custom membranes for molecular simulations. *J. Chem. Theory Comput.* **11**, 2144–2155 (2015). 26574417 10.1021/acs.jctc.5b00209

[54] Abraham, M. J. et al. GROMACS: high performance molecular simulations through multi-level parallelism from laptops to supercomputers. *SoftwareX* **1-2**, 19–25 (2015).

[55] Bussi, G., Donadio, D. & Parrinello, M. Canonical sampling through velocity rescaling. *J. Chem. Phys.* **126**, 014101 (2007). 17212484 10.1063/1.2408420

[56] Berendsen, H. J. C. et al. Molecular dynamics with coupling to an external bath. *J. Chem. Phys.* **81**, 3684–3690 (1984).

[57] Santos, D. E. S. et al. SuAVE: a tool for analyzing curvature-dependent properties in chemical interfaces. *J. Chem. Inf. Model.* **60**, 473–484 (2020). 31508962 10.1021/acs.jcim.9b00569

[58] Lolicato, M. et al. Transmembrane helix straightening and buckling underlies activation of mechanosensitive and thermosensitive K_2P_ channels. *Neuron* **84**, 1198–1212 (2014). 25500157 10.1016/j.neuron.2014.11.017 PMC4270892

[59] Lian, X. J. et al. Directed cardiomyocyte differentiation from human pluripotent stem cells by modulating Wnt/β-catenin signaling under fully defined conditions. *Nat. Protoc.* **8**, 162–175 (2013). 23257984 10.1038/nprot.2012.150 PMC3612968

[60] Sala, L. et al. Use of hiPSC-derived cardiomyocytes to rule out proarrhythmic effects of drugs: the case of hydroxychloroquine in COVID-19. *Front. Physiol.* **12**, 730127 (2022). 35153806 10.3389/fphys.2021.730127 PMC8829511

