# Supplementary material for: Membrane-targeted push-pull azobenzenes for the optical modulation of membrane potential
Source: Light Sci Appl. 2025 Jan 1;14:8. doi: 10.1038/s41377-024-01669-x (PMC11688454; doi:10.1038/s41377-024-01669-x)
Supplement: Supplementary file 1 — Supplementary Material [file 41377_2024_1669_MOESM1_ESM.docx]

Membrane-targeted push-pull azobenzenes for the optical modulation of membrane potential

Valentina Sesti^1,2#^, Arianna Magni^2,3#§^, Matteo Moschetta^2#^, Chiara Florindi^2,4^, Marlene E. Pfeffer^5^, Mattia Lorenzo DiFrancesco^6^, Michele Guizzardi^3^, Giulia Folpini^2,7^, Luca Sala^4,8^, Alessandra Gilda Ritacca^5,9^, Beatrice Campanelli^5,9^, Paola Moretti^1,2^, Giuseppe Maria Paternò^2,3^, Luca Maragliano^5,9^, Matteo Tommasini^1^, Francesco Lodola^2,4^, Elisabetta Colombo^5,6^, Fabio Benfenati^5,6^, Chiara Bertarelli^1,2*^ & Guglielmo Lanzani^2,3^*

1. Department of Chemistry, Materials and Chemical Engineering “Giulio Natta” Politecnico di Milano, Milano, 20133, Italy
2. Center for Nanoscience and Technology, Istituto Italiano di Tecnologia, Via Rubattino 81, 20134, Milano, 20134, Italy
3. Department of Physics, Politecnico di Milano, Milano, 20133, Italy
4. Department of Biotechnology and Biosciences, University of Milano-Bicocca, Milano, 20126, Italy
5. Center for Synaptic Neuroscience and Technology, Istituto Italiano di Tecnologia, Largo Rosanna Benzi 10, Genova, 16132, Italy
6. IRCCS Ospedale Policlinico San Martino, Largo Rosanna Benzi 10, Genova, 16132, Italy
7. Institute for Photonics and Nanotechnologies (IFN), National Research Council (CNR), Piazza Leonardo da Vinci, 32, 20133, Milano, Italy
8. Istituto Auxologico Italiano IRCCS, Center for Cardiac Arrhythmias of Genetic Origin and Laboratory of Cardiovascular Genetics, Milano, 20095, Italy
9. Department of Life and Environmental Sciences, Polytechnic University of Marche, Via Brecce Bianche, Ancona, 60131, Italy

§ Present address: Department of Materials Science and Engineering, Stanford University, Stanford, CA, 94305, USA

#These authors contributed equally to this work

*Correspondence to be addressed to Chiara Bertarelli ([chiara.bertarelli@polimi.it](mailto:chiara.bertarelli@polimi.it)) and Guglielmo Lanzani ([guglielmo.lanzani@polimi.it](mailto:guglielmo.lanzani@polimi.it))

SUPPLEMENTARY INFORMATION

# Supplementary Materials and Methods

**Synthesis and Chemical Characterization**

**Synthesis of 1.** 4-(4-Nitrophenylazo)aniline , commercially knows as Disperse Orange 3 (4.13 mmol, 1 equiv.) is added to a solution of potassium carbonate (8.26 mmol, 2 equiv.) and 1,6-dibromohexane (4.21 mmol, 1.2 equiv.) are subjected to three vacuum-argon cycles and then stirred in 20mL of anhydrous acetonitrile. The mixture is kept stirring at 60 °C for 120 h under Ar..The raw product is purified by flash chromatography over silica gel using dichloromethane: hexane with a ratio of 4:1 as eluent. The recovered product 1 is dried under vacuum obtaining a red solid with a 34% yield.

δ _H_ (400 MHz, DMSO) 8.37(d, 2H, J=9.2 Hz, Ph), 7.94(d, 2H, J=9.1 Hz, Ph), 7.85(d, 2H, J=9.2 Hz, 7.08 (s, 1H, -NH), 6.85(d, 2H, J=9.3 Hz, Ph), 3.17(t, 2H, -CH2NH), 3.55(t, 2H, -CH2Br), 1.91-1.25(m, 8H).

**Synthesis of MTP1.** 1 (0.12 mmol) is dissolved in 7mL of pyridine and left stirring for 120 h at room temperature. The excess of pyridine is then removed under vacuum (10^-1^ mbar, 60 °C).and he final product MTP1 is then washed with hexane to further remove traces of pyridine. MTP1 is obtained as an orange solid in quantitative yield.

δ _H_ (400 MHz, DMSO) 9.09 (2 H, d, *J* 5.7, pyr), 8.61 (1 H, t, *J* 8.0, pyr), 8.36 (2 H, d, *J* 9.1, Ph), 8.16 (2 H, t, *J* 7.1, pyr), 7.91 (2 H, d, *J* 9.1, Ph), 7.79 (2 H, d, *J* 9.0, Ph), 7.07 (1 H, t, *J* 5.3, NH), 6.72 (2 H, d, *J* 9.0, Ph), 4.61 (2 H, t, *J* 7.4, -CH_2_pyr), 3.20 – 3.13 (2 H, m), 1.98 – 1.92 (2 H, m), 1.63 – 1.55 (2 H, m), 1.46 – 1.40 (2 H, m), 1.34 (2 H, m) ^13^C NMR (101 MHz, DMSO) δ 156.30, 153.57, 146.65, 145.45, 144.71, 142.98, 128.05, 126.39, 124.92, 122.30, 111.75, 60.68, 42.19, 30.61, 28.13, 25.86, 25.13.

MS: 404.3 (M-1Br)+

1H-NMR spectrum of MTP1

13C-NMR spectrum of MTP1


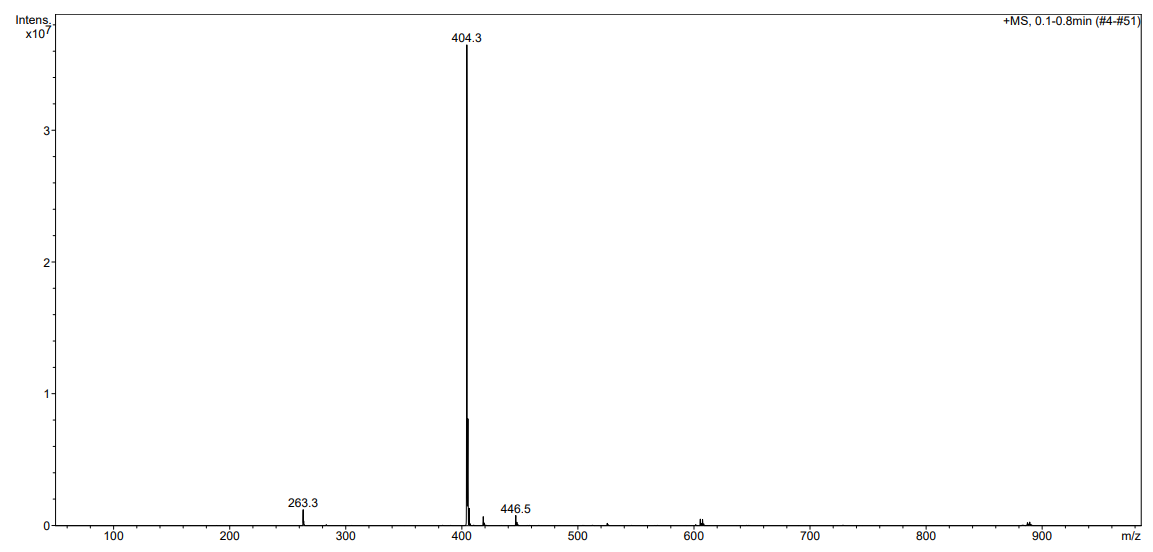


ESI-MS spectrum of MTP1

**Synthesis of MTA1**. Trimethylamine is added dropwise to 1(0.1 mmol, 1 equiv.) in 7 mL of ethanol. The reaction bath is left stirring at 80 °C for 48 h. After removing the solvent under reduced pressure (10^-1^ mbar, 60 °C) the resulting product MTA1 is washed with small portions of hexane. MTA1 is obtained as an orange solid in quantitative yield.

δ _H_ (400 MHz, DMSO) 8.36 (2 H, d, *J* 9.1, Ph), 7.91 (2 H, d, *J* 9.1, Ph), 7.80 (2 H, d, *J* 9.0, Ph), 7.09 (1 H, t, *J* 5.7, NH), 6.74 (2 H, d, *J* 9.1, Ph), 3.45(2H, m), 3.19 (3 H, m), 3.04 (9 H, s), 1.77 - 176 (2 H, m), 1.65 – 1.58 (2 H, m), 1.47 – 1.41 (2 H, m), 1.38 – 1.30 (2 H, m). ^13^C NMR (101 MHz, DMSO) δ 156.29, 153.58, 146.68, 146.48, 143.00, 126.41, 124.94, 122.31, 65.24, 52.14, 42.21, 28.19, 25.97, 25.50, 21.99.

MS: 384.3 (M-1Br)+

1H-NMR spectrum of MTA1

13C-NMR spectrum of MTA1


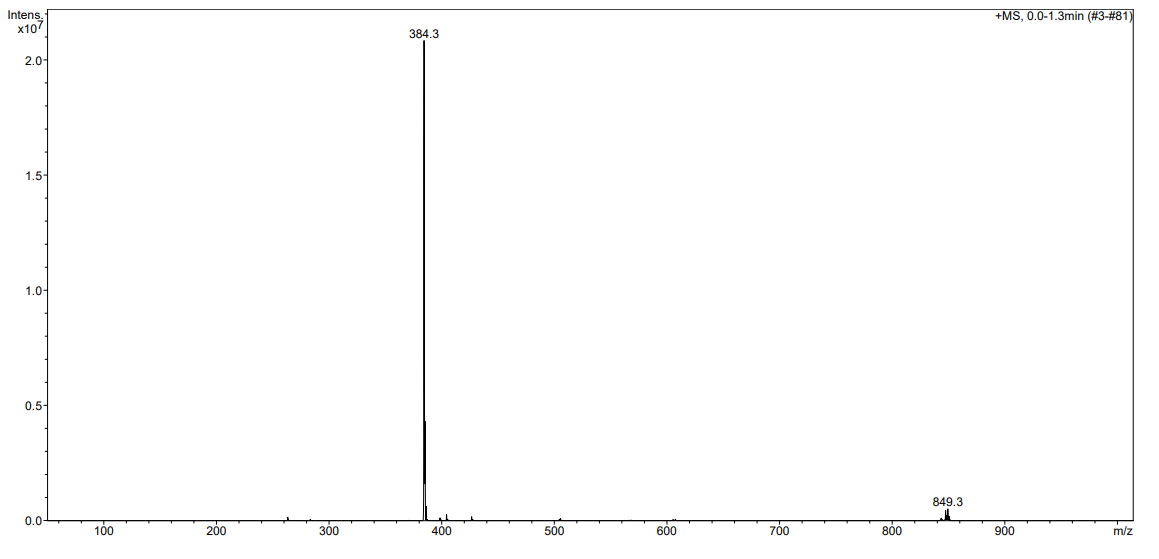


ESI-MS spectrum of MTA1

**Synthesis of 2-3.** A mixture of aniline (16.4 mmol, 1 equiv.) and dibromohexane (47.8 mmol, 2.92 equiv.) is left stirring for 10 h at 95 °C under inert atmoshpere, and then left cooling to room temperature. Subsequently, water is added to the resulting mixture, stirred for 10 min, then extracted with ethyl acetate and dried over sodium sulfate. After solvent removal under reduced pressure, the raw product is purified by flash column chromatography in silica gel using hexane/dichloromethane with a ratio of 2,5:1 as eluent. Compound 2 is obtained with a 60% yield as a yellow oil while compound 3 is obtained with 34% yield.as a pale-yellow oil.

Compound 2: ^1^H NMR(400 MHz, CDCl_3_) δ 7.14(m, 2H, Ph), 6.56(m, 3H, Ph), 3.35(t, 4H, J=6.8 Hz, -CH2Br), 3.18(t, 4H, J=7.4 Hz, -CH2N), 1.83-1.76(m, 4H), 1.56-1.48(m, 4H), 1.45-1.37(m, 4H), 1.31-1.24(m, 4H)

Conmpound 3: ^1^H NMR(400 MHz, CDCl_3_) δ 7.15(m, 2H, Ph), 6.68(m, 3H, Ph), 3.45 (t, J = 6.8 Hz, 2H, -CH2Br), 3.14 (t, J = 7.1 Hz, 2H, -CH2N), 1.95-1.84 (m, 2H), 1.72-1.60 (m, 2H), 1.57-1.40 (m, 2H).

**Synthesis of 4.** 4-nitroaniline (10 mmol, 1 equiv.) is dissolved in 100 mL of tetrahydrofuran. It is cooled to -20 °C and then boron trifluoride diethyl etherate (15 mmol, 1.5 equiv.) is added dropwise, followed by tertbutyl nitrite (12 mmol, 1.2 equiv.). This mixture is left stirring for 1 hour at -20°C and then is allowed to warm up to room temperature. Finally, the precipitate is collected on a Büchner funnel and washed with tetrahydrofuran to obtain compound 4 with a 95% yield as a white solid.

^1^H NMR(400 MHz, MeOD) δ 8.9(d, 2H, J=9.4 Hz, 2.5Hz), 8.75 (d, 2H, J=9.4 Hz, 2.5 Hz)

**Synthesis of 5.** A solution of 4 (0.48 mmol, 1 equiv.) in 3 mL of acetonitrile is added dropwise to a solution of N,N-bis(6-bromohexyl)benzenamine (0.48 mmol, 1 equiv.) in 20 mL of acetonitrile at 5 °C over 3 h while stirring. The solution is left stirring for 12 hours to warm at room temperature and, finally, a solution of potassium carbonate (0.48 mmol, 1 equiv.) in 2 mL of water is added. After solvent removal under reduced pressure, the crude product is purified by flash column chromatography in silica gel using dichloromethane/hexane with a ratio 3:5 as eluent. 5 is obtained as a red solid with a 46% yield.

^1^H NMR(400 MHz, DMSO) δ 8.36(d, 2H, J=9.1 Hz, Ph), 7.93(d, 2H, J=9.1 Hz, Ph), 7.84(d, 2H, J=9.2 Hz, Ph), 6.85(d, 2H, J=9.3 Hz, Ph), 3.54(m, 4H, -CH2Br), 3.43(m, 4H, -CH2N), 1.89-1.28 (m, 16H)

**Synthesis of MTP2.** 4 mL of pyridine is added to 5 (0.06 mmol, 1 equiv.). The reaction bath is left stirring at room temperature for 24 h. After removing the solvent under reduced pressure, the resulting product is washed with small portions of hexane multiple times, leading to a red solid in quantitative yield.

δ _H_ (400 MHz, DMSO) 9.22 (4 H, d, *J* 5.8, pyr), 8.62 (2 H, t, *J* 7.8, pyr), 8.33 (2 H, d, *J* 9.0, Ph), 8.17 (4 H, t, *J* 7.0, pyr), 7.90 (2 H, d, *J* 9.0, Ph), 7.80 (2 H, d, *J* 9.1, Ph), 6.81 (2 H, d, *J* 9.2, Ph), 4.68 (4 H, t, *J* 7.3, -CH_2_pyr), 3.41 (4 H, t, *J* 7.1, -CH_2_N), 2.01 – 1.86 (4 H, m), 1.55 (4 H, m), 1.34 (8 H, m). ^13^C NMR (101 MHz, DMSO) δ 156.25, 151.62, 146.63, 145.46, 144.75, 142.41, 128.03, 126.18, 124.94, 122.36, 111.48, 60.51, 50.16, 30.65, 26.65, 25.22, 24.59.

MS: 646 (M-1Br)+

1H-NMR spectrum of MTP2

13C-NMR spectrum of MTP2

C
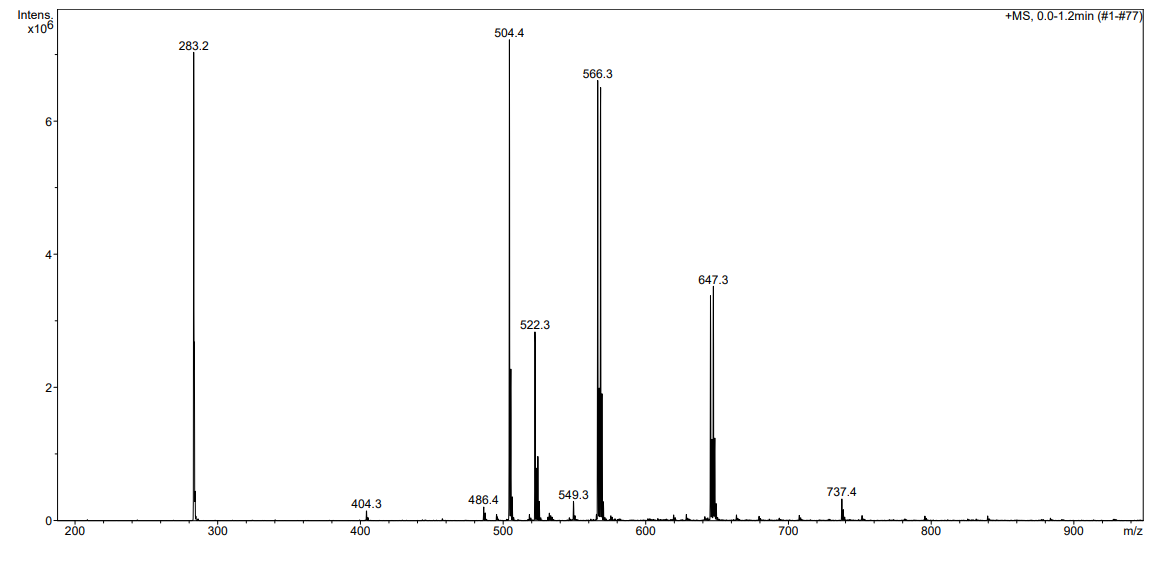


ESI-MS spectrum of MTP2

**Synthesis of MTA2.** 0.3 mL of trimethylamine is added dropwise to 5 (0.06 mmol, 1 equiv.) dissolved in 4 mL of ethanol. The reaction bath is left stirring at 80 °C for 48 h. After solvent removal under reduced pressure, the resulting product is washed with small portions of hexane to lead to a red solid in quantitative yield.

δ _H_ (400 MHz, DMSO) 8.37 (2 H, d, *J* 9.0, Ph), 7.91 (2 H, d, *J* 8.8, Ph), 7.84 (2 H, d, *J* 9.2, Ph), ii6.85 (2 H, d, *J* 9.4), 3.48 – 3.40 (4 H, m), 3.25 (4 H, m), 3.03 (18 H, s), 1.69 (4 H, s), 1.62 (4 H, s), 1.33 (8 H, m). ^13^C NMR (101 MHz, DMSO) δ 156.27, 151.67, 146.77, 142.49, 126.18, 124.98, 122.37, 111.48, 65.23, 52.16, 50.15, 26.69, 25.77, 25.68, 22.02.

MS: 605 (M-1Br)^+^

^^

1H-NMR spectrum of MTA2

^^

13C-NMR spectrum of MTA2

^
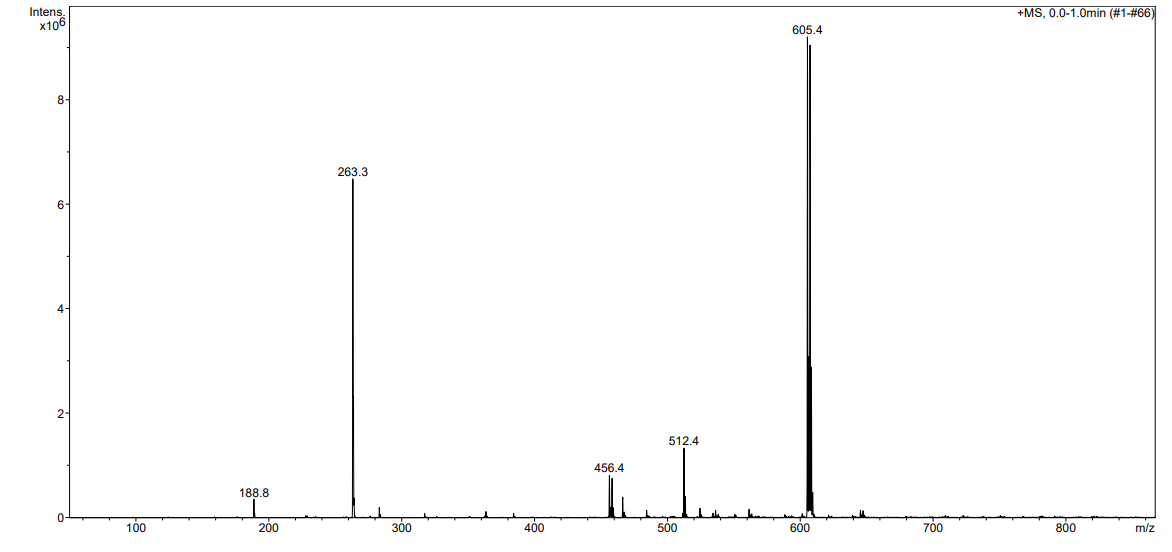
^

ESI-MS spectrum of MTA2

The purity of the final samples prepared was checked by means of CHNS analysis and verified to be over 95% for all samples: specifically, from the calculated versus found masses we could calculate the percentage error in the masses:

|  |  | **C** | **H** | **N** | **S** |
| --- | --- | --- | --- | --- | --- |
| **MTP1** | found | 56.32 | 5.49 | 14.32 | -- |
|  | calc | 57.03 | 5.41 | 14.46 | -- |
|  | err % | 1.24 | 1.48 | 0.97 | -- |
| **MTA1** | found | 51.61 | 6.82 | 14.86 | -- |
|  | calc | 54.31 | 6.51 | 15.08 | -- |
|  | err % | 4.97 | 4.76 | 1.48 | -- |
| **MTP2** | found | 55.94 | 5.84 | 11.52 | -- |
|  | calc | 56.21 | 5.83 | 11.57 | -- |
|  | err % | 0.48 | 0.17 | 0.43 | -- |
| **MTA2** | found | 52.02 | 7.19 | 12.11 | -- |
|  | calc | 52.48 | 7.34 | 12.24 | -- |
|  | err % | 0.88 | 2.04 | 1.06 | -- |

Indicating a purity of 98.52% for MTP1, 95.03% for MTA1, 99.52% for MTP2 and 98.04% for MTA2, respectively. Purity of the samples was assessed through elemental analysis performed on a CHNS analyzer Costech. The percentage difference in mass between the theoretical and experimental mass was used to determine the purity grade of the compounds.

**DFT calculations.** We have carried out B3LYP/6-31+G(d,p) DFT calculations, including D3BJ empirical dispersion, on molecular models of Ziapin2 and MTP2 that represent the photoactive azobenzene core and the characteristic electroactive groups. The dipoles have been computed for the fully geometry-optimized cis and trans isomers of both molecules.

# Supplementary Figures


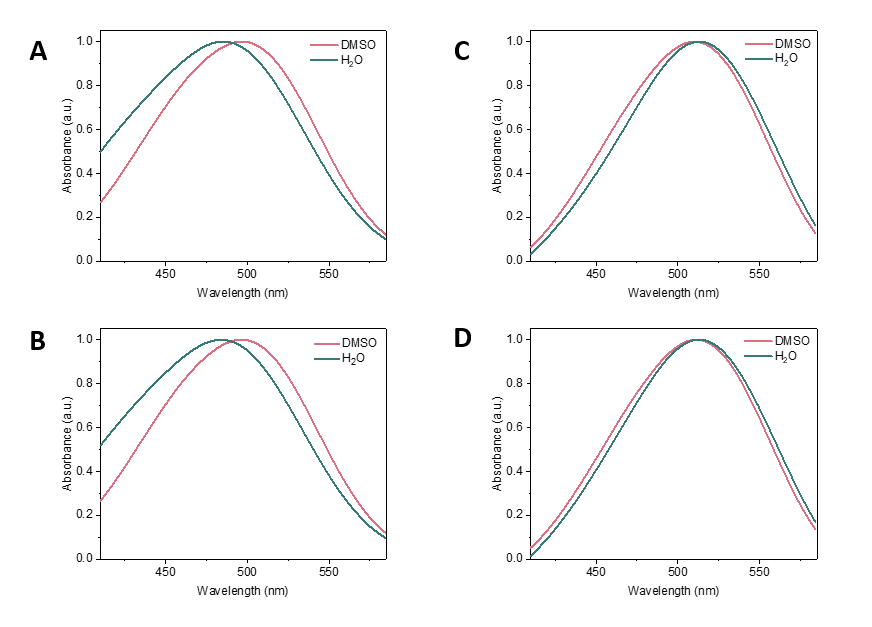


| **Table S1.** Absorption peak wavelength and extinction coefficient for MTs in DMSO and water. | | | | |
| --- | --- | --- | --- | --- |
|  | $\boldsymbol{\lambda}_{\boldsymbol{max}}$ **in DMSO**  **(nm)** | **ε in DMSO**  **(M^-1^cm^-1^)** | $\boldsymbol{\lambda}_{\boldsymbol{max}}$**in H_2_O**  **(nm)** | **ε in H_2_O**  **(M^-1^cm^-1^)** |
| **MTP1** | 497 | 27900 | 485 | 29400 |
| **MTA1** | 496 | 27400 | 483 | 28800 |
| **MTP2** | 511 | 27500 | 513 | 30300 |
| **MTA2** | 511 | 27700 | 513 | 29700 |

**Figure S1.** UV-vis absorption spectra of A. **MTA1**, B. **MTP1**, C. **MTA2** and D. **MTP2** in DMSO and water. On the bottom, the calculated molar absorption coefficients (ε) of each molecule in the two solvents.


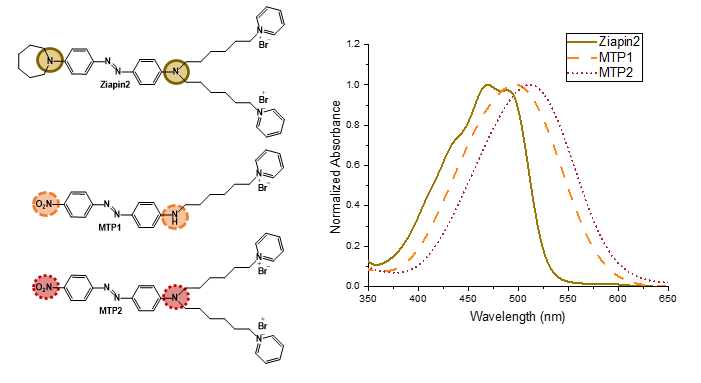


**Figure S2.** UV-Vis spectra of Ziapin2, MTP1 and MTP2 in DMSO.


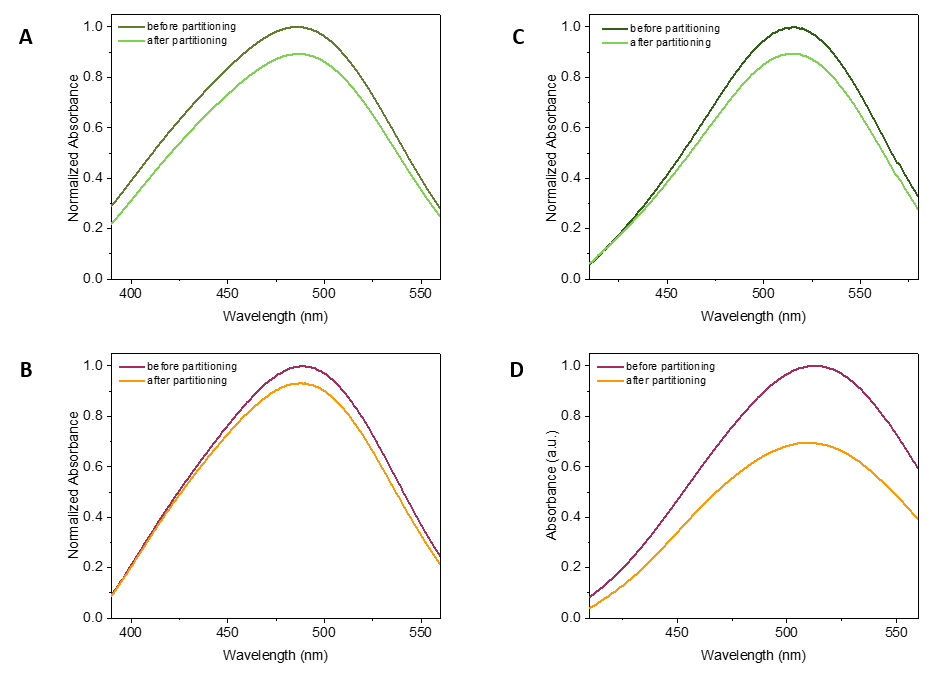


**Figure S3. UV-Vis absorption spectra of the four molecules in the cell media before and after partitioning** A. MTA1, B. MTP1, C. MTA2 and D. MTP2**.** The biological experiments were carried out on HEK293T cells. Firstly, MTs were diluted in the extracellular medium (KRH) at the final concentration of 10 µM, and the UV-vis absorption spectra were recorded as a reference. The solution was then put in contact with HEK293T cells for 5 min. After the incubation, the solution was collected and UV-Vis spectra were recorded and normalized to the absorption maximum of the naive azobenzenes. The difference in absorbance was evaluated as an estimation of the amount of MTs that have been successfully associated with cells. MTP2 revealed a significant drop in absorbance after the partitioning that is three times stronger than the other MTs (8%, 10%, 11% and 30% for MTP1, MTA1, MTA2 and MTP2, respectively), suggesting that has a stronger affinity of MTP2 for the cell membrane.


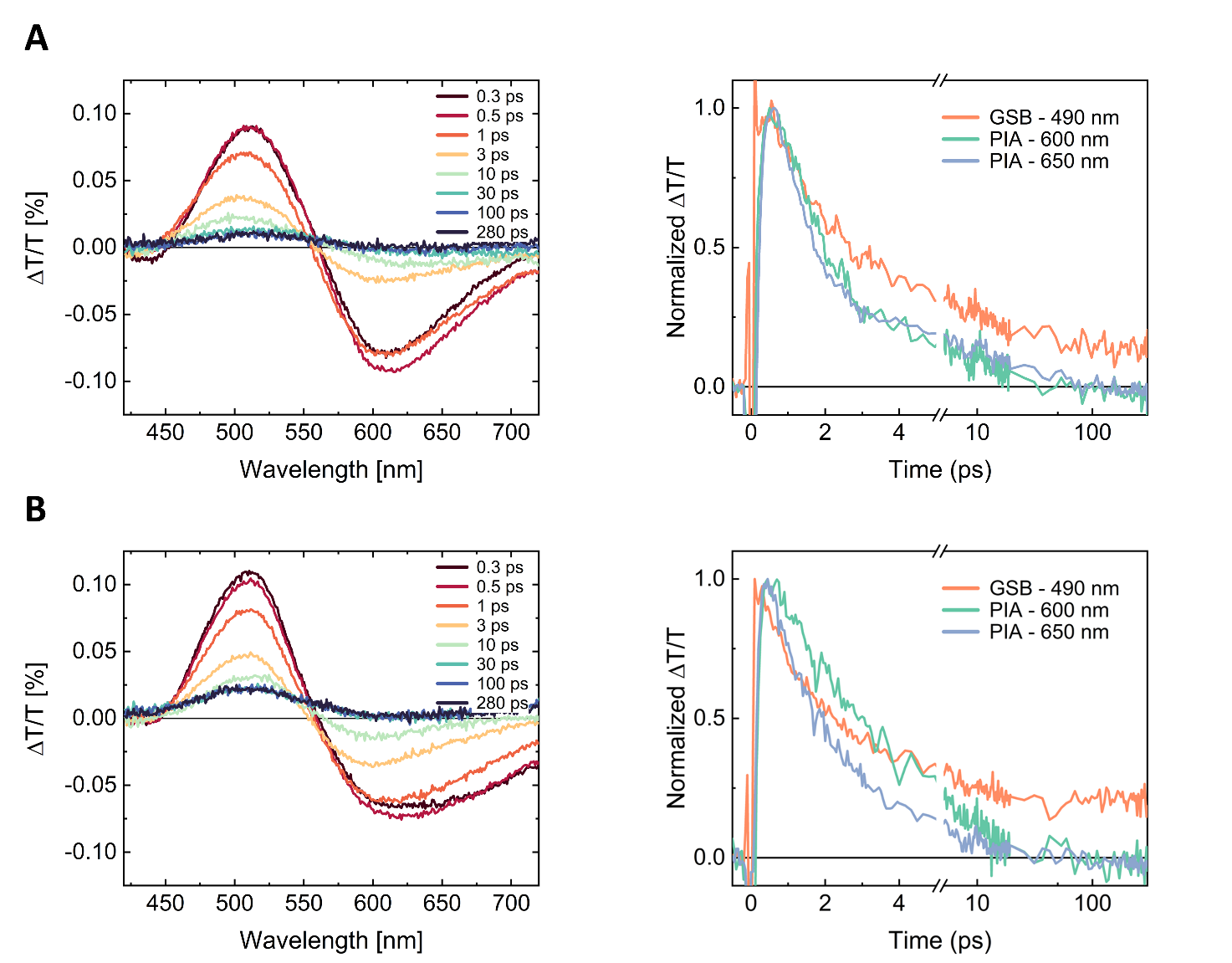


**Figure S4.** **Ultrafast** t**ransient absorption spectroscopy of MTP2 [25 µM] in water and SDS micelles suspension.** Differential transmission spectra and dynamics of MTP2 in water (A) and SDS micelles suspension (B). The system was excited at 500 nm and probed with sapphire-generated white light super-continuums in the visible range. The solutions were flowed using a peristaltic pump, to avoid photobleaching and accumulation of cis isomer.


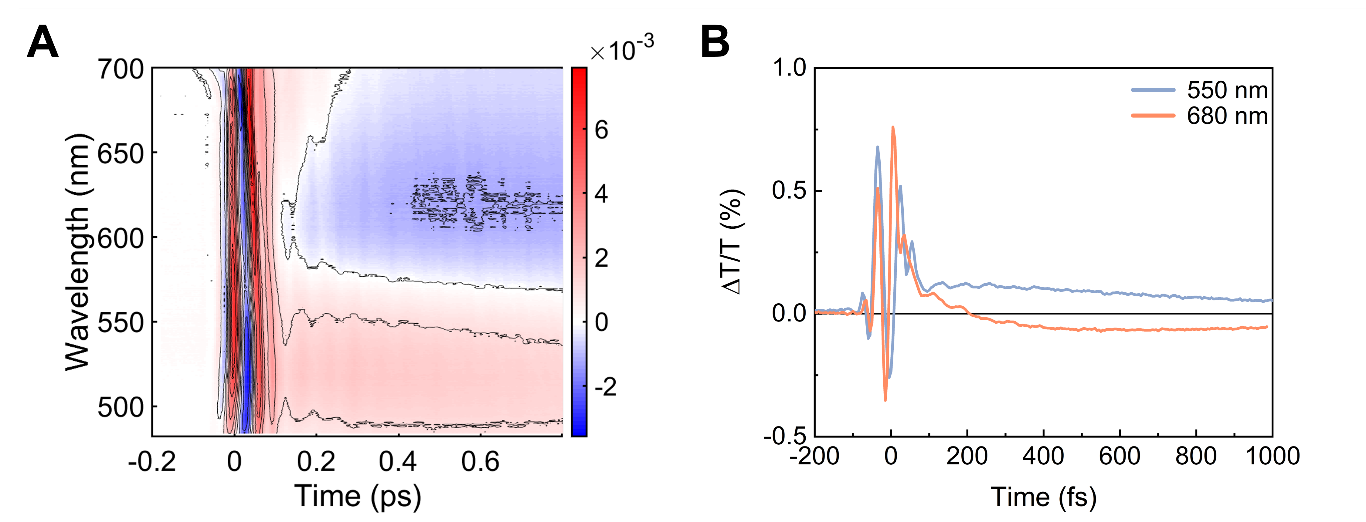


**Figure S5.** **Sub-ps transient absorption spectroscopy of MTP2 [25 µM] in water.** Differential transmission map (A) and dynamics (B) of MTP2 in water [25 μM]. The sample was pumped with a broadband optical pulse of around 30 fs to resolve the fast system relaxation dynamics. The solution was flowed using a peristaltic pump, to avoid photobleaching and accumulation of cis isomer. The map displays a positive (red) feature above 650 nm, which disappears in around 300 fs. This positive peak is a stimulated emission (SE) signal coming from the excited state reached immediately after the excitation, i.e., S_1_ in the trans geometry (S_1, TRANS_). The SE signal disappears very rapidly, likely due to a conformational relaxation of the molecule that hampers the radiative decay. Estimating the lifetime of S_1, TRANS_ is complex, as the positive SE signal overlaps not only with the negative one coming from the two photoinduced absorption bands, but also with cross-phase modulation artefacts and coherent oscillations related to the solvent.


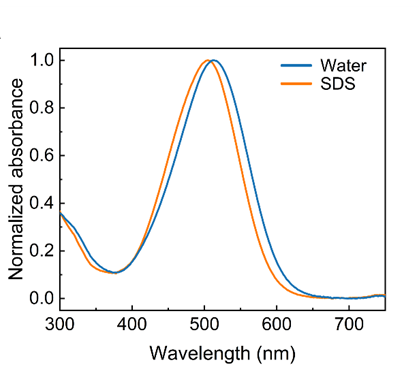


**Figure S6. Steady-state spectroscopy on MTP2 in water and SDS micelles** **suspension.** Absorption spectra of MTP2 [25 μM] in water and SDS [100 mM] micelles suspension.


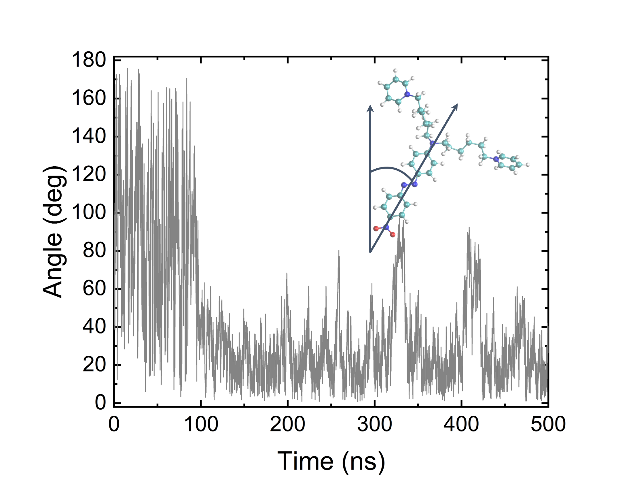


**Figure S7.** Time-dependence of the angle formed by the main molecular axis of MTP2 and the bilayer normal along one of the five trajectories in Figure 2B.


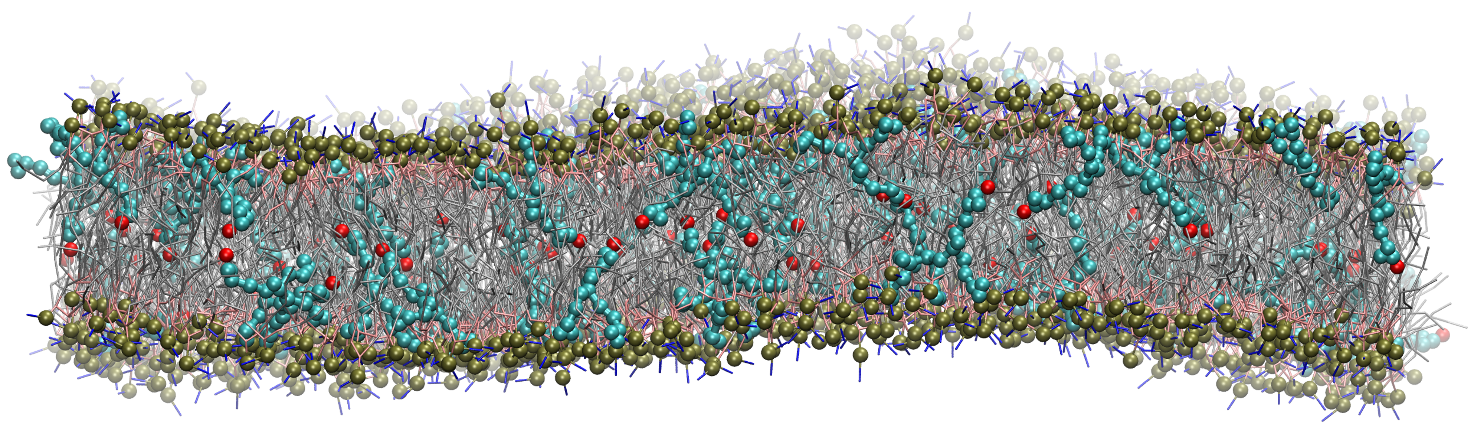


**Figure S8**. Snapshot from the CG-MD simulation of 120 MTP2 molecules (cyan) in a POPC membrane; the red spheres represent the nitro-groups.


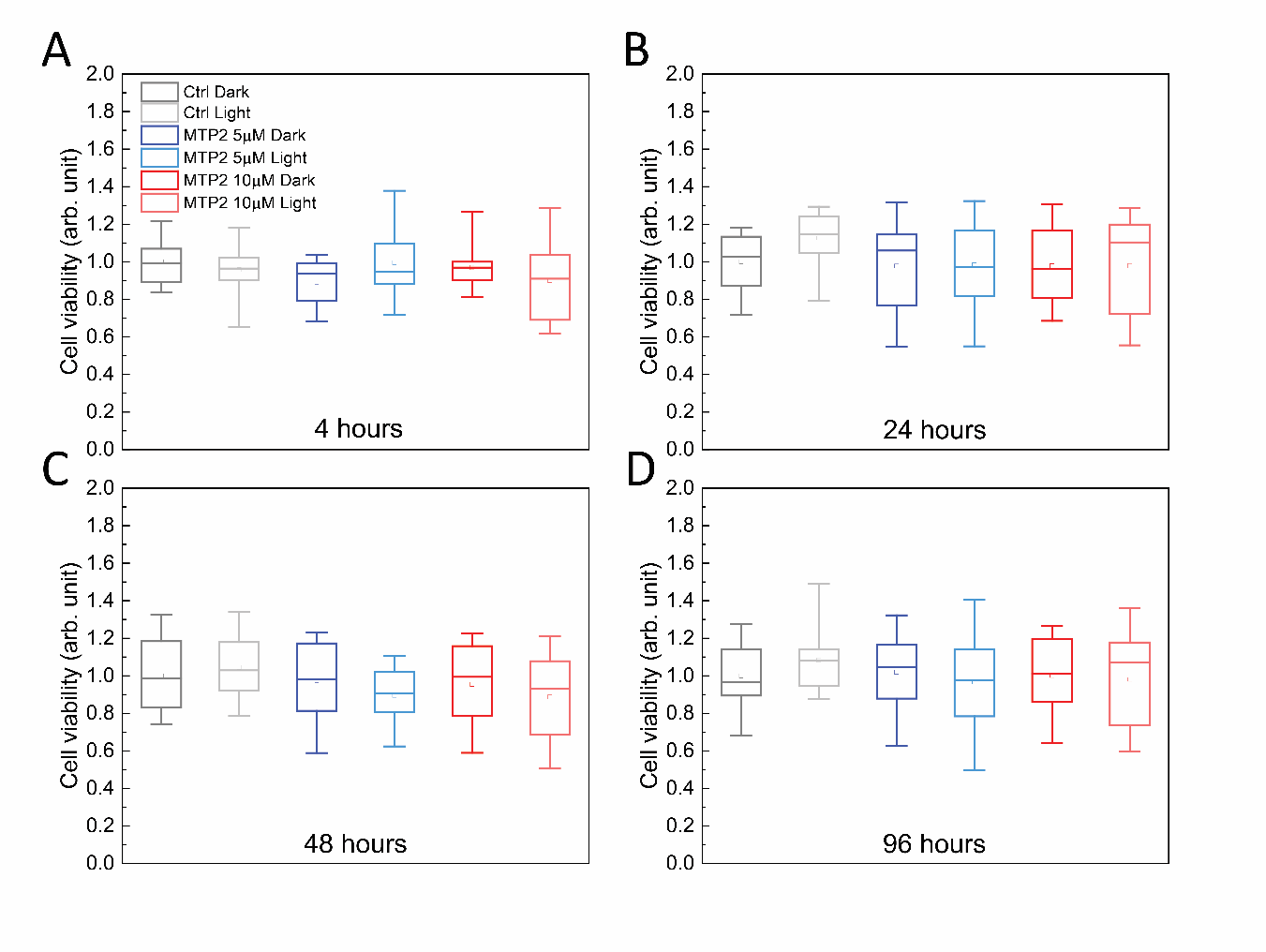


**Figure S9. MTP2 does not affect the viability of HEK293T cells.** A-D. Box plots representing viability of HEK293T cells loaded with 5 and 10 µM MTP2 for 5 min. Half of the samples were stimulated with light at 200 µWmm^-2^ for 30 s. Cell viability was analysed at different times, as described in the Materials and Methods. Cell viability of Ctrl in the dark was set to 1. p>0.05, two-way ANOVA for repeated measures/Bonferroni’s tests (n=12 per each condition).


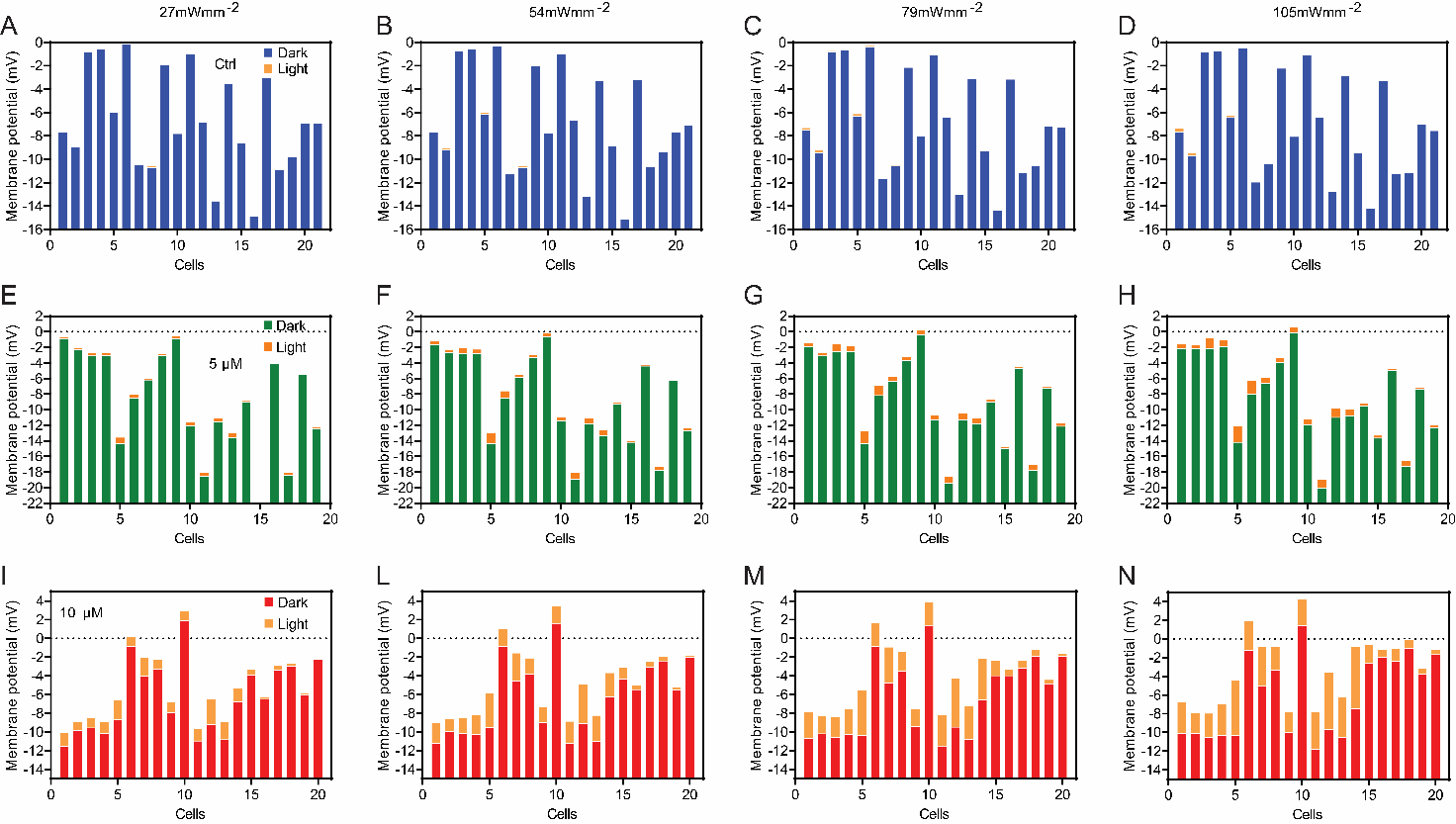


**Figure S10. Membrane potential absolute values before and after the application of light.** Membrane potential values under dark conditions (colour) and after 20ms of light irradiation (yellow) per each single recorded HEK293T cell are shown. Ctrl cells are represented in blue (A-D), cells treated with 5µM of MTP2 are represented in green (E-H) and cells treated with 10µM of MTP2 are represented in red (I-N).


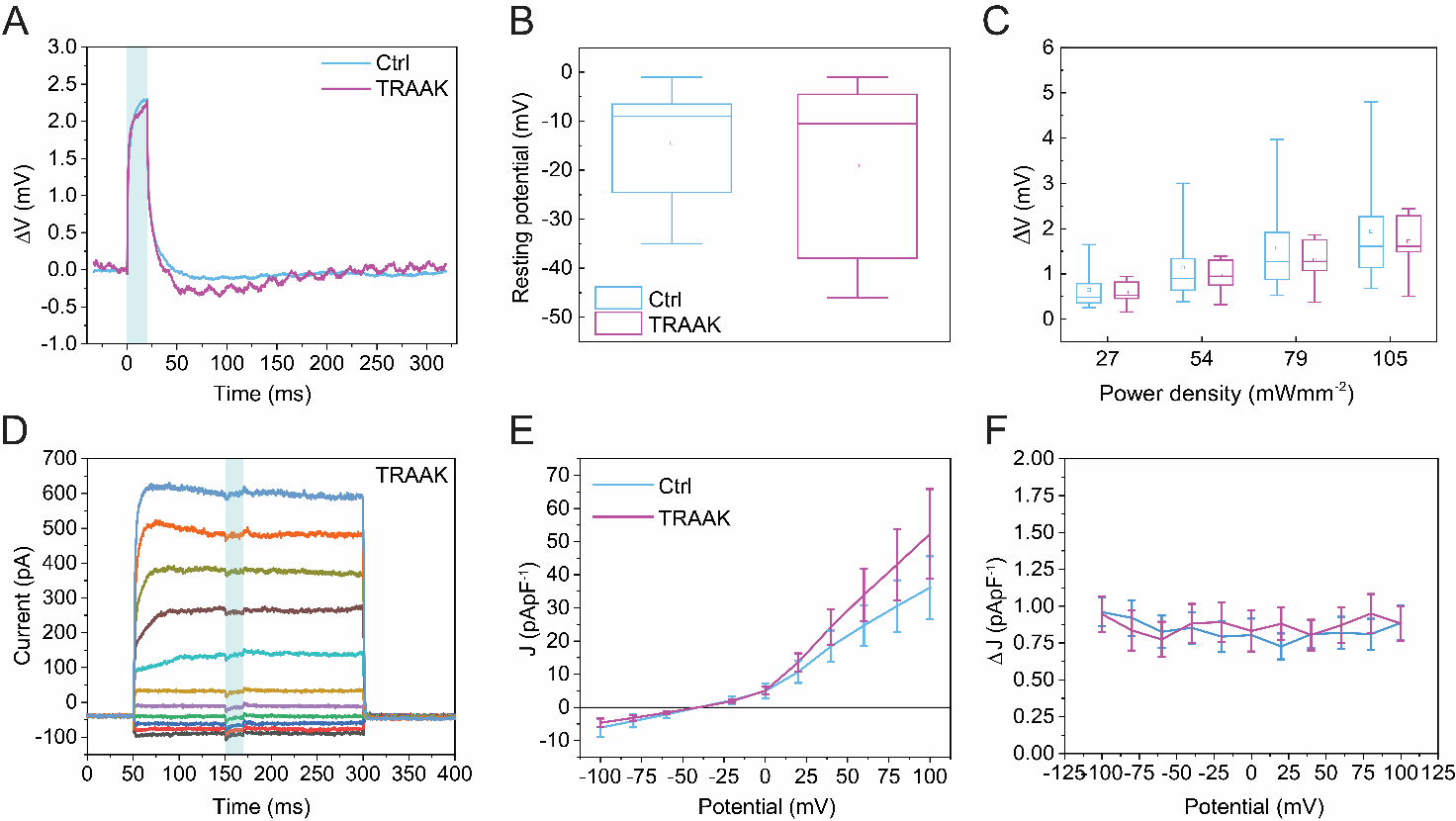


**Figure S11. Expression of mechano-sensitive channels does not affect light-dependent membrane potential modulation.** A. Representative whole-cell current clamp traces recorded from HEK293T cells either untreated (CTRL, blue) or transfected with the mechano-sensitive channel TRAAK (violet). Cells were loaded with 10 µM of MTP2 for 5 min and illuminated at 105 mWmm^-2^ for 20 ms. B,C. Box plots representing the resting membrane potential (B) and the peak depolarization (C) in HEK293T cells treated as in A. In C., cells were exposed at different power densities (27, 54, 79 and 105 mWmm^-2^). p>0.05; unpaired Student’s t-test or Mann Whitney’s U-test (n=8 for each condition). D. Representative whole cell voltage clamp trace recorded from TRAAK-transfected HEK293T cells incubated for 5 min in the presence of 10 µM MTP2 and illuminated for 20 ms at 105 mWmm^-2^. The voltage step protocol is from -100mV to 100mV with a step of 20mV. E-F. Graphs representing the current peak under dark conditions (E) and during illumination (F) from HEK293T cells either untreated (CTRL, blue) or transfected with the mechano-sensitive channel TRAAK (violet). Data are shown as mean ± sem. P>0.05; two-way ANOVA/Bonferroni’s tests. (n=12 for each condition).
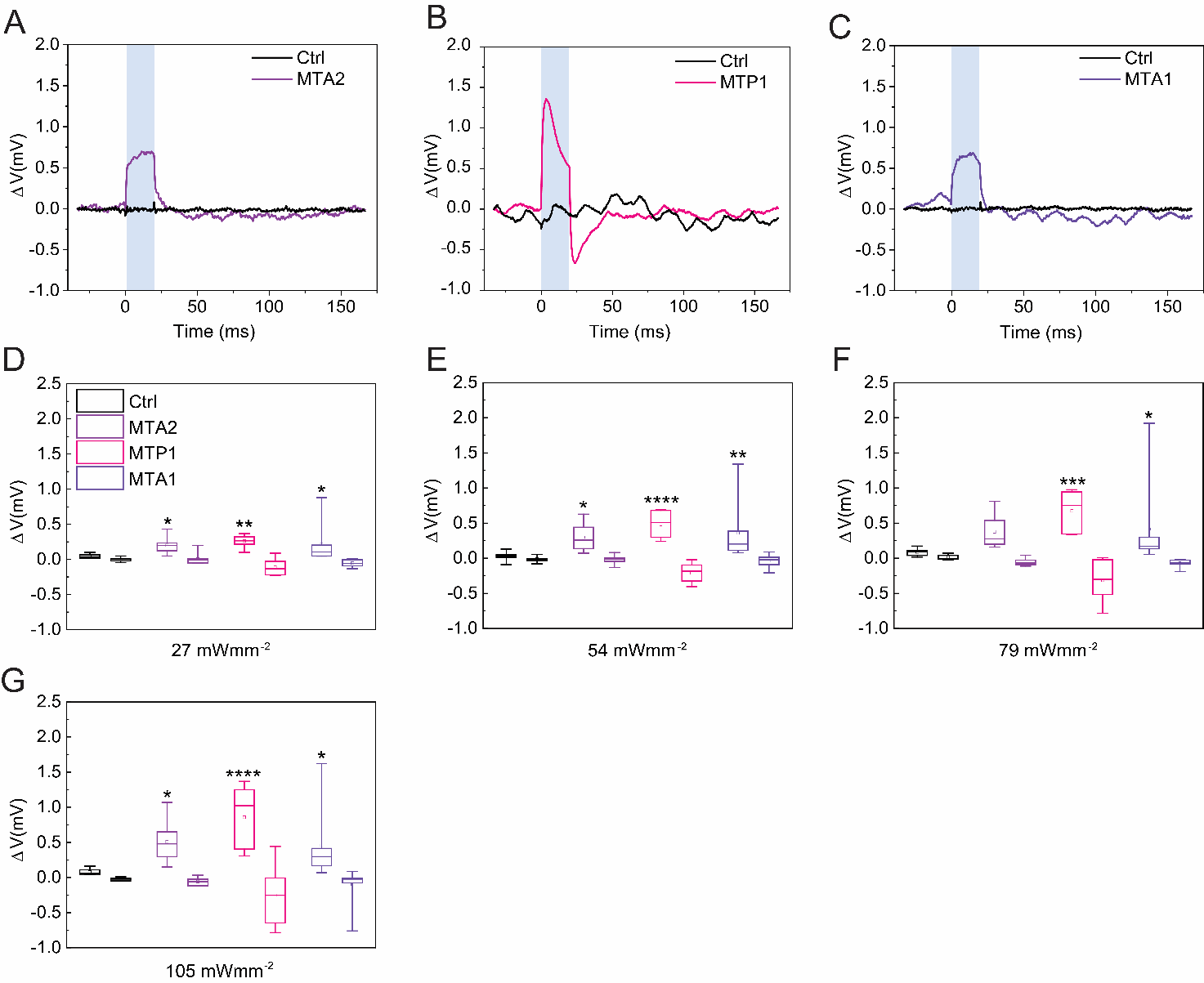


**Figure S12. Light-evoked membrane potential changes by MTA2, MTP1 and MTA1.** A-C. Representative whole-cell current clamp traces recorded from HEK293T cells loaded with vehicle (Ctrl, black trace), MTA2, MTP1 or MTA1 (10 µM; color traces) for 5 min at 105 mWmm^-2^. D-G. Box plots of peak depolarization and peak hyperpolarization in HEK293T cells subjected to 20 ms of light stimulation in the absence (Ctrl) or presence of MTA2, MTP1 or MTA1 at different power densities (27, 54, 79 and 105 mWmm^-2^). *p<0.05, **p<0.01, ***p<0.001 and ****p<0.0001; two-way ANOVA/Dunnett’s test vs Ctrl (n=9, 8, 7 and 7 for Ctrl, MTA2, MTP1 and MTA1, respectively).

*
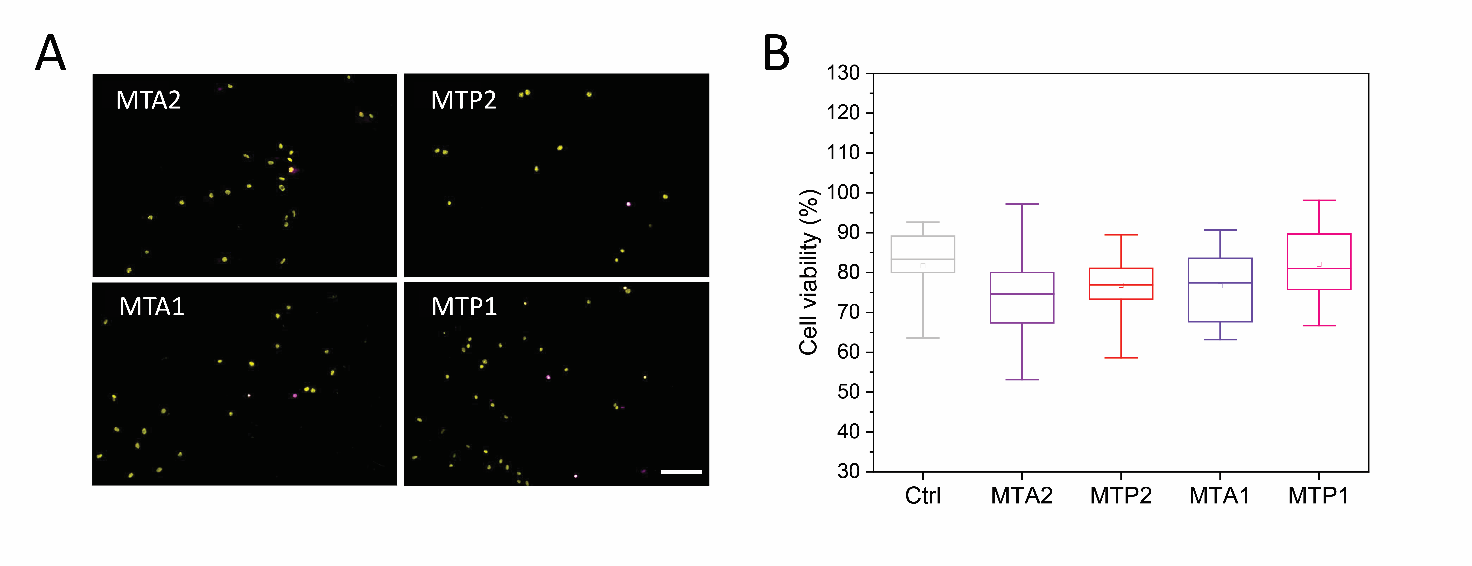
*

***Figure S13.* *MTA2, MTP2, MTA1 and MTP1 do not affect the viability of primary hippocampal neurons.*** *A. Representative images of fluorescence microscopy for the viability test on primary hippocampal neurons incubated with the battery of MTs (5 μM). All neurons are stained with Hoechst (yellow) and apoptotic cells with propidium iodide (purple). Scale bar, 100 μm. B. Viability after incubation either in the vehicle (Ctrl, grey box) or with the different MT molecules (5 μM; color boxes). Cell viability was evaluated from the percent ratio (All cells - Propidium iodide-positive cells) / All cells). P>0.05; one way-ANOVA/Tukey’s multiple comparison test (n=23, 14, 17, 15 and 14 for Ctrl, MTA2, MTP2, MTA1 and MTP1, respectively).*


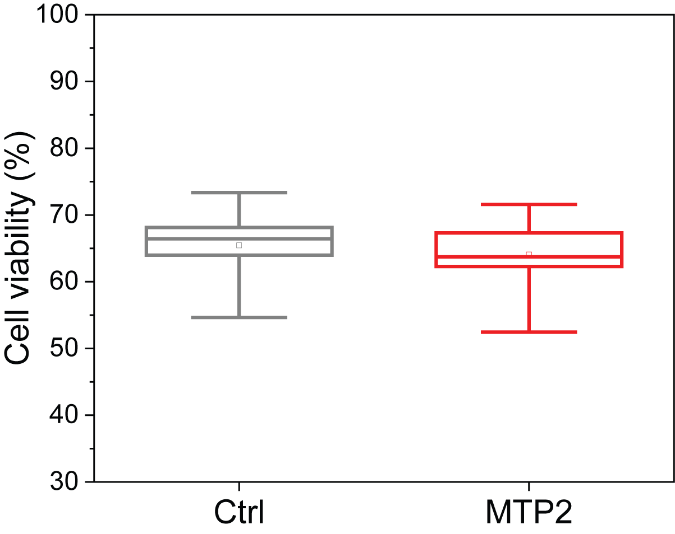


**Figure S14.** Cell viability of primary hippocampal neurons at DIV 14, after incubation either in vehicle (Ctrl) or with MTP2 10 μM. Percent cell viability was calculated from the ratio of DAPI positive nuclei and Propidium iodide signals from permeabilized neurons (All cells - Propidium iodide-positive cells) / All cells * 100). P>0.05; unpaired t-test (n=13 per each condition from 2 distinct neuronal preparations).


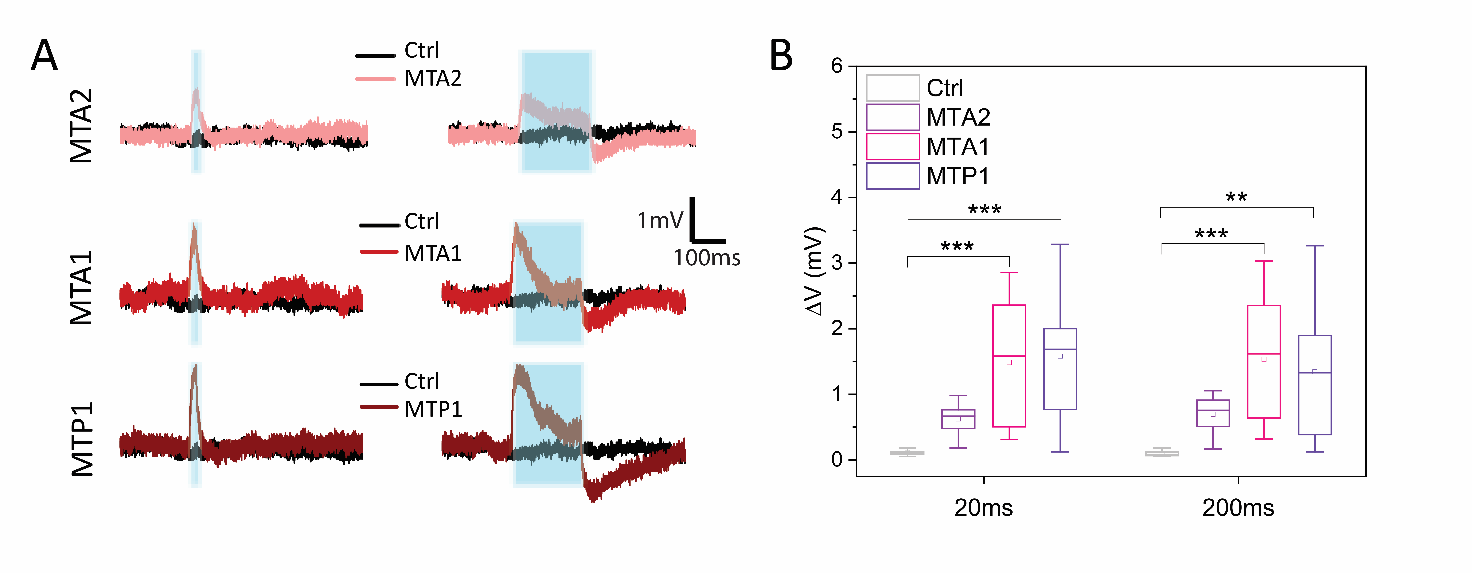


**Figure S15. Light-evoked membrane potential changes by MTA2, MTA1 and MTP1.** A. Representative whole-cell current clamp traces recorded from primary hippocampal neurons loaded with vehicle (Ctrl, black trace), MTA2, MTP1 or MTA1 (5 µM; color traces) for 5 min. B. Box plots of peak depolarization of primary hippocampal neurons subjected to 20 ms of light stimulation in the absence (Ctrl) or presence of MTA2, MTP1 or MTA1 at 20 mWmm^-2^. **p<0.01 and ***p<0.001; one-way ANOVA/Dunnett’s test vs Ctrl (n=9, 8, 11 and 10-13 for Ctrl, MTA2, MTA1 and MTP1, respectively).


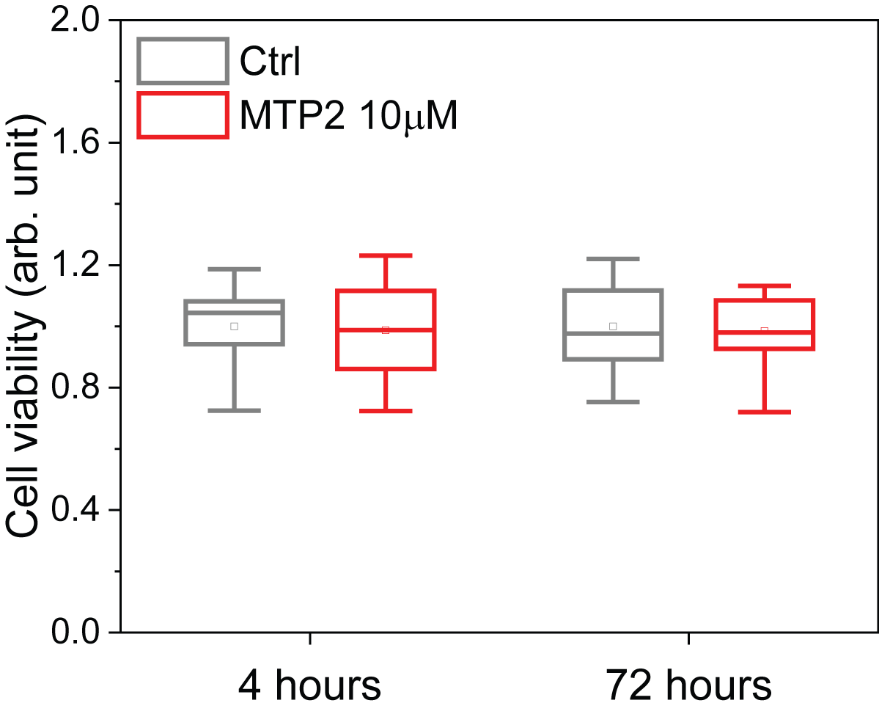


**Figure S16. MTP2 does not affect the viability of hiPSC-CMs.** Box plots representing viability of hiPSC-CMs loaded with 10 µM MTP2 for 5 min (in red) and relative controls (in grey). Cell viability was analysed at both 4 and 72 hours after the incubation. Cell viability of Ctrl was set to 1. p>0.05, Unpaired Student’s t test (n=16 per each condition and time point).


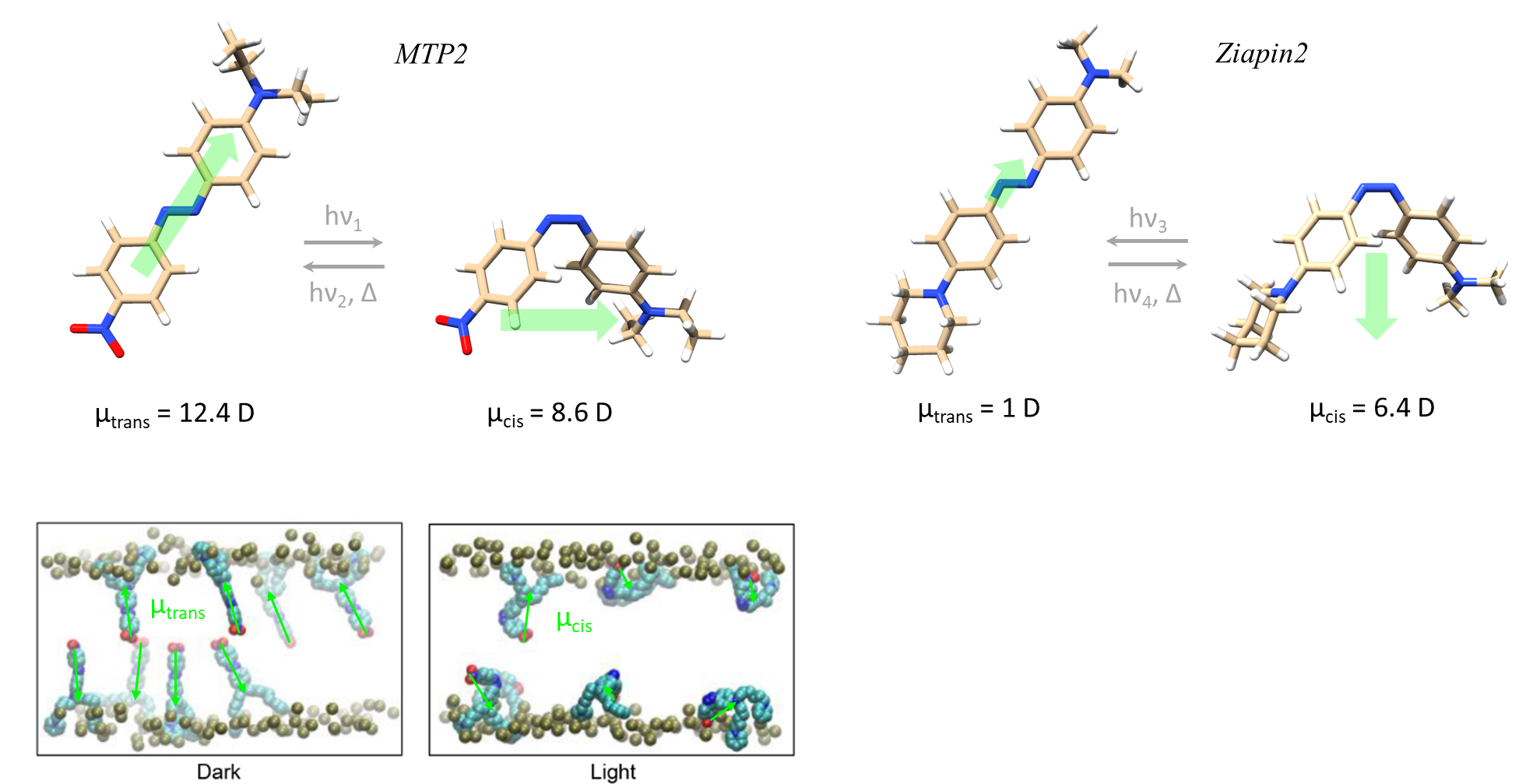


**Figure S17.** (top panels) DFT calculation of the trans to cis variation of the dipole moment of representative molecular models of MTP2 (left) and Ziapin2 (right). Green arrows show the direction of the dipole on the molecular structures. The two bottom panels are taken from Figure 2C of the main text (snapshot from molecular dynamics) and show the orientation of the MTP2 dipoles in the membrane.


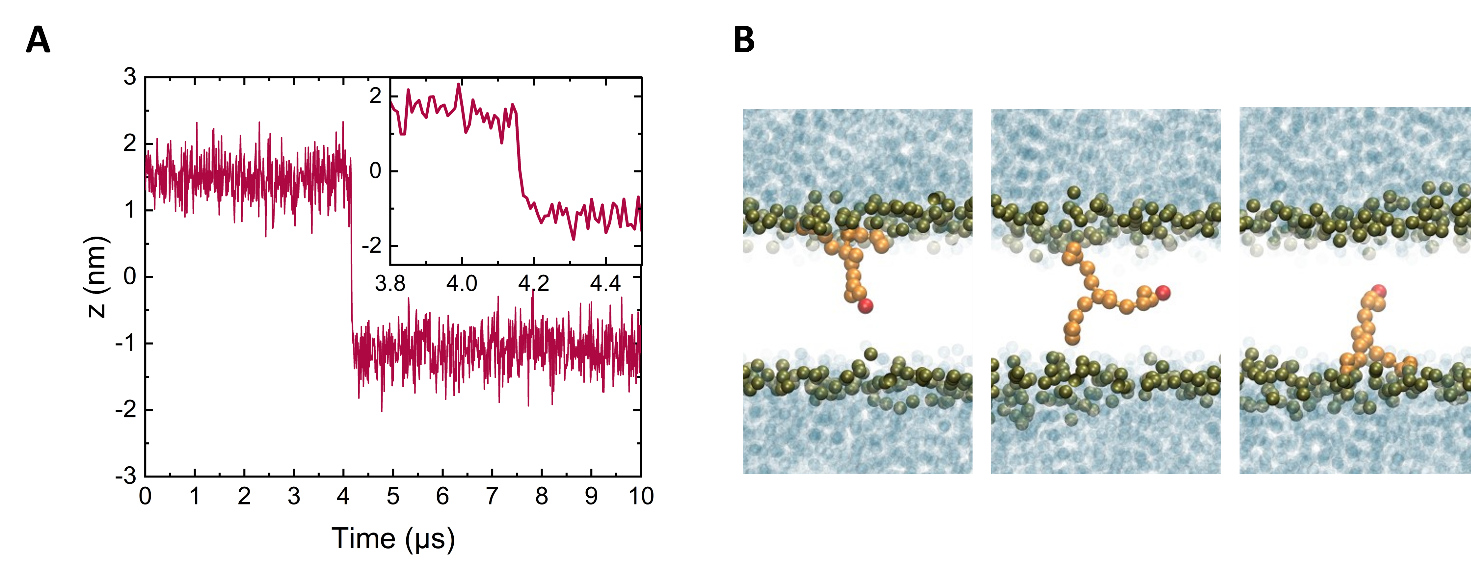


**Figure S18. The transmembrane voltage promotes MTP2 flip-flop events in CG-MD simulations.** A. Time dependence of the z-coordinate of a flipping MTP2 pyridine bead; the inset shows a close-up view of the transition. B. Snapshots representing three consecutive moments of a flip-flop event. The red sphere represents the nitro group, while the blue spheres are water molecules.

# Supplementary Text

## From the photoisomerization to the dynamic of the RC equivalent circuit

The effects driven by the photoisomerization of MTP2 in cells can be included in the electrical equivalent RC circuit model of a cell.


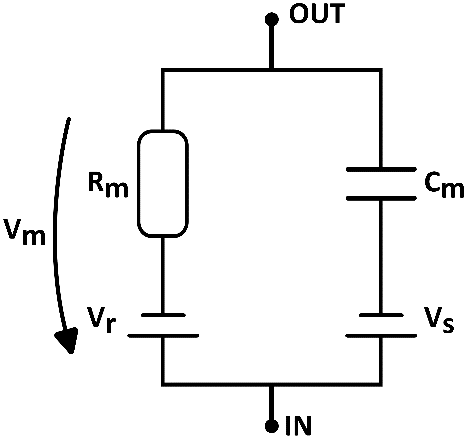


The capacitor $C_{m}$ accounts for the electrical insulation and charge accumulation provided by the membrane hydrophobic core of the lipid bilayer that lies between the intra- and extra-cellular media, which are two electrolytes and thus conductive media. The conductance of ion channels, together with the intrinsic membrane permeability, are represented by the resistor $R_{m}$.

The presence of charged groups in the phospholipids’ heads produces a surface charge, which generates a surface potential on each side of the membrane. The asymmetry in the composition of the two membrane leaflets, the selective permeability of the membrane and the ionic concentration of the electrolytes are responsible for an asymmetric surface charge distribution and thus a potential surface difference between the internal and external edges of the membrane, which is represented by the battery $V_{s}$ in the equivalent circuit. Note that, under resting conditions, $V_{s}$ is related to the resting membrane potential $V_{r}$ and the potential drop on the membrane $V_{t}$ by $V_{s}=V_{r}-V_{t}$.

The resistor $R_{m}$ is in series with the battery, whose potential $V_{r}$ is given by the equilibrium potential of the conducting ions, described by the Goldman-Hodgkin-Katz equation. The upper and lower nodes of the circuit represent the extracellular and intracellular fluids, respectively. The membrane potential is given by the voltage difference at these two nodes, $V_{m}=V_{in}-V_{out}$.

In this framework, it is fundamental to link the variation of the molecule dipole moment to a change in one of the parameters of the equivalent RC circuit. The photoinduced dipole moment $\Delta\vec{\mu}$ induces a displacement of charges in the neighbourhood of the cell membrane. According to the model of the cell membrane as a plane capacitor, the variation of the dipole moment will reflect in a change of the charges stored on the two plates of the capacitor, thus altering the potential drop across the capacitor itself. The dipole moment $\Delta\vec{\mu}$ induces the accumulation of positive charges at the outer plate of the capacitor and negative charges at the inner one. Going back to the equivalent RC circuit, this causes a positive variation of the surface potential $V_{s}$. Finally, assuming that $V_{s}$ is the only parameter altered by the photoinduced variation of MTP2, the RC circuit temporal evolution is described by the differential equation:

$$\frac{\partial V_{m}}{\partial t}=\frac{\partial V_{s}}{\partial t}-\frac{V_{m}-V_{r}}{C_{m}R_{m}}$$

To estimate the light-driven change in $V_{s}$, or at least its order of magnitude, we suppose that each cell internalizes 10^8^ molecules of MTP2. This is a reasonable estimation as usually cells are plated on glass slides so that at the time of exposure to the push-pull molecule, there are around 3,000,000 cells on each glass slide. These samples are then covered with 1 mL solution of MTP2 at the concentration of 10 μM. We assume that ~ 30% of the MPT2 molecules are present, with ~ 50% of them distributed to the membrane. Out of the 10^8^ molecules, we only take into account about 10% of them, specifically the excess fraction of molecules that can give rise to a net dipole moment change. We then presume that all the molecules undergo the trans→cis photoisomerization, resulting in a dipole moment variation of *Δμ* = 1.25 × 10^−29^ Cm. As the cell membrane has an average thickness of 4 nm, we take this length as the distance between the charges of the dipole, which gives $q$ = 3.1 × 10^−21^ C as the charge separated by each MTP2 molecule. Assuming that each cell has a membrane capacity $C_{m}$ of 25 pF, the surface potential change ${\Delta V}_{s}$ can then be computed as

${\Delta V}_{s}={10}^{7}\frac{q}{C_{m}}\cong$ 4 mV.

This rather simple estimation conducted to a photoinduced variation of $V_{s}$ in the order of a few millivolts, which is a reasonable value, in agreement with the measured amplitude of the membrane depolarization signal. Clearly, a more precise estimation of the amplitude of $\Delta V_{s}$depends on the precise number of molecules that lies within the cell and their distribution among the two membrane leaflets, which is also affected by intrinsic biological variability. Moreover, the isomerization kinetics depends on both the membrane status and its fluidity, and the illumination intensity employed in the stimulation protocol. Variations $\Delta V_{s}$ are subjected to a non-linear dependence on the illumination intensity. We assume $\Delta V_{s}$ to be proportional to the number of molecules in the *cis* form.

How the *cis* population varies can be examined by considering the isomerization process. When an ensemble of MTP2 molecules is placed under illumination photoisomerization reactions take place and a number of molecules in the thermodynamically stable *trans* state pass to the metastable cis state and *vice versa*. Being $n_{trans}$ and $n_{cis}$ the fraction of molecules in the *trans* and *cis* state, respectively, the isomerization process is described by the following equation.

$$\frac{d n_{trans}}{dt}=-k_{TC}I\left( t \right)n_{trans}\left( t \right)+k_{CT}I\left( t \right)n_{cis}\left( t \right)+\gamma n_{cis}\left( t \right)$$

Here, $\gamma$ is the rate of the cis to trans thermal relaxation. $k_{TC}$ and $k_{CT}$ are the photo-isomerization rate constants, for the trans to cis and cis to trans processes, respectively, i.e. the absorption cross-section normalized by the photon energy. I is the actinic light intensity. In dark conditions, i.e. $I = 0$, the population dynamics reduces to:

$$\frac{dn_{trans}}{dt}=\gamma n_{cis}\left( t \right)$$

Considering an ensemble of MTP2 molecules, all in the trans state, subjected to a light stimulus, switched on at time $t$ = 0 with intensity $I_{0}$, the population of the two isomers will reach a steady state condition, where the ratio between cis and trans population is given by

$$\frac{n_{cis}}{n_{trans}}=\frac{k_{TC}I_{0}}{k_{CT}I_{0}+\gamma}$$

and the fraction of the population in cis configuration will be:

$$n_{cis}=\frac{k_{TC}I_{0}}{{{(k}_{TC}+k}_{CT})I_{0}+\gamma}$$

The build-up of the cis population will occur with a characteristic time constant $\tau_{build-up}=\frac{1}{\left( k_{CT}+k_{TC} \right)I_{0}+\gamma}$. On the other hand, the relaxation, which happens as light offset, proceeds with $\tau_{relax}=\gamma^{-1}$ as the characteristic time constant. These time constants rule the cell photostimulation process.

The surface potential change is then given by

$$\Delta V_{s}\left( I \right)=\Delta V_{s,0}n_{cis}$$

where $\Delta V_{s,0}$ is a parameter that corresponds to maximum change (all molecules in cis).

As far as the kinetics of the process is concerned, we suppose $V_{s}$ varies exponentially.

$$V_{s}\left( t \right)=V_{s,0}+\Delta V_{s}\left( I \right)\left[ \left( 1-\exp\left( -\frac{t-t_{0}}{\tau_{build-up}} \right) \right)H\left( t-t_{0} \right)-\left( 1-\exp\left( -\frac{t-t_{0}-\Delta t}{\tau_{relax}} \right) \right)H\left( t-t_{0}-\Delta t \right) \right]$$

Here, $H\left( x \right)$ is the Heaviside function, which is equal to zero for $x < 0$ and equal to 1 otherwise and $\Delta t$ is the light pulse duration, which is 20 ms, as in the experiments.

The simulations are run using the parameters reported in **Table S2**.

| **Table S2.** Parameters used to simulate the cell response to MTP2 photoisomerization as a function of the light intensity. | |
| --- | --- |
| **Parameter** | **Value** |
| $V_{s}$ | 100 mV |
| $\Delta V_{s,0}$ | 2 mV |
| $R_{m}$ | 3 G$\Omega$ |
| $C_{m}$ | 25 pF |
| $V_{r}$ | -25 mV |
| $k_{TC}$ | 0.006 mm^2^ mW^-1^ ms^-1^ |
| $k_{CT}$ | 0.001 mm^2^ mW^-1^ ms^-1^ |
| $\gamma$ | 1 ms^-1^ |

The cell parameters were defined considering reasonable values for HEK293T cells, while the time constants were chosen to nicely reproduce the membrane voltage modulation kinetics retrieved from the experimental data. The results of the computed membrane potential modulation are shown in **Figure 8**. The model replicates the characteristic features of the experimental data. Increasing the light intensity employed for the stimulation, both the depolarization and the hyperpolarization peak amplitudes grow. Note that the kinetics of the depolarizing signal are influenced by light intensity, as the higher this parameter the faster the depolarization peak is reached. This aspect agrees with the experimentally observed behaviour, which is found by looking at the representative traces in **Figure 4**. Moreover, if the light intensity *I* is sufficiently high, the amplitude of $\Delta V_{s,0}$, and therefore of the depolarization peak, becomes constant, as a sort of saturation is reached. However, the saturation is not realized within the intensity range adopted in the experiments.
